# A scoping review of feed interventions and livelihoods of small-scale livestock keepers

**DOI:** 10.1038/s41477-020-00786-w

**Published:** 2020-10-12

**Authors:** Isabelle Baltenweck, Debbie Cherney, Alan Duncan, Erin Eldermire, Edda Tandi Lwoga, Ricardo Labarta, Elizaphan James Oburu Rao, Steven Staal, Nils Teufel

**Affiliations:** 1 International Livestock Research Institute, Nairobi, Kenya; 2 Cornell University, Ithaca, NY USA; 3 University of Edinburgh, Edinburgh, UK; 4 College of Business Education, Dar es Salaam, Tanzania; 5 International Center for Tropical Agriculture (CIAT), Cali, Colombia

**Keywords:** Developing world, Scientific community, Social sciences

## Abstract

Livestock support the livelihoods of one billion people in Africa, Asia and Latin America, but the productivity of animals remains low, reducing the potential of the sector to support higher incomes and better nutrition. Improved livestock feeding has been identified as the most important step towards higher productivity. This scoping review assessed the evidence for the uptake of improved ruminant livestock feed options, the effect of this uptake on livestock productivity and the degree to which this improves smallholder farmer livelihoods. In total, 22,981 papers were identified, of which 73 papers were included in the final analysis after a rigorous double-blind screening review. Only papers that reported farmers’ decision to use a new feed intervention were selected, thereby excluding feeding trials and participatory feed assessments. Of the 73 papers, only 6 reported combined evidence of adoption, effect on productivity and livelihood changes. A total of 58 papers looked at adoption, 19 at productivity change and 22 at livelihood change. This scoping review highlights the gap in evidence for the adoption of new livestock feeding practices and provides recommendations to support farmers’ uptake of feed interventions.

## Abstract (web summary)

Livestock support the livelihoods of a billion people in Africa, Asia and Latin America, and improved feeding could increase productivity. However, this scoping review highlights serious gaps in evidence around the adoption of new feeding practices.

## Main

One billion poor people in Low- and Middle-Income Countries (LMICs) derive part of their livelihood from livestock^1^. As well as providing income and financial security for the rural poor, livestock products are a vital source of protein and micronutrients in regions where their regular supply could reduce the currently high prevalence of childhood stunting^2^. Unlike in the Global North, in LMICs, urbanization and growth in incomes and populations are fuelling strong growth in the demand for livestock products. A key challenge therefore is to identify and promote solutions to increase livestock production and productivity. Currently, livestock productivity is much lower in LMICs than in high-income countries^3^. For example, the average cow milk yields in Western Europe are 20 times higher than in Eastern Africa^4^. Moreover, increasing per-animal productivity is essential for environmental sustainability as systems intensify. For example, sub-Saharan Africa is a livestock greenhouse gas emissions intensity hotspot mainly because yields are low^5^. Poor productivity is caused by many factors including unimproved genetic stock, inadequate veterinary provision and a general scarcity of high-quality inputs. Although access to enough high-quality feed is generally agreed to be the main constraint on better productivity^6,7^, the livestock feed challenge has proved to be intractable despite considerable research and development effort over the past three decades. This effort has included many programmes aimed at delivering high-quality feed options to smallholder livestock keepers, but the evidence for their effectiveness is sparse^8^. The research field is characterized by anecdotes with very little rigorous and systematic analysis of success and the factors underlying success^9^.

Feeding practices are varied and comprise the mix of different feeds offered to livestock (planted forages, crop residues such as straw, supplementation with commercial feeds and so on) and the amounts offered to different animals in different seasons. Feed improvement interventions among LMIC smallholders include the introduction of improved grasses and legumes^10^, the use of multipurpose trees^11^, methods of increasing intake and the nutritive value of crop residues by physical or chemical treatment^12^, and methods of preserving fresh feed to fill seasonal feed gaps^13^ (Box 1). Although ‘improved’ feed options have been researched and promoted widely in many systems by many stakeholders over many years^14^, less research has addressed the uptake of these options by farmers, their effects on ruminant productivity and ultimately their impact on farmer livelihoods, possibly due to overall underinvestment in impact assessment studies^9^. The objective of this scoping review is to assess the availability and quality of evidence for the uptake of improved livestock feed options by small-scale producers, the effects of this uptake on ruminant livestock productivity and the degree to which this improves smallholder farmer livelihoods (Box 2). The overarching aim is to identify promising strategies to improve livestock feeding for improved productivity and ultimately farmer livelihoods, on the basis of the analysis of the reviewed evidence.

**Table 1:** Numbers of studies in different categories by type of feed intervention

Categories	Items in category	Total	Planted forages	Agroforestry	Crop residues
Publication type	Peer-reviewed journal article	51	35	17	4
	Book chapter	1	1	1	
	Conference proceeding	8	6	3	1
	Report	6	6	2	1
	Working paper	7	5	3	1
Year of publication	2016–2019	15	13	3	
	2011–2015	21	15	3	4
	2001–2010	28	18	14	3
	Before 2001	9	7	6	
Agro-ecological zone	Mixed systems	53	35	21	6
	Agro-pastoral systems	10	8	3	
	Pastoral systems	3	3		
	Multiple systems	1	1	1	1
	Other	2	2		
	(Blank)	4	4	1	
Region	Horn of Africa	12	11	3	
	East Africa	23	16	9	3
	Central Africa	1	1		
	West Africa	7	4	2	1
	Southern Africa	5	1	3	1
	South Asia	6	4	2	2
	Southeast Asia	12	11	3	
	East Asia	2	2	1	
	Latin America	5	3	3	
Type of methods	Quantitative	45	31	18	5
	Qualitative	9	6	4	
	Quantitative/qualitative	19	15	4	2
Duration of the experiment	>20 years	3	1	2	
	11–20 years	4	2	2	1
	1–5 years	22	17	8	1
	6–10 years	8	7	3	
	NA	36	26	11	5
**Total**		**73**	**53**	**26**	**7**

NA, not applicable.

**Table 2:** Numbers of studies in different categories by level along the impact pathway

Categories	Items in category	Total	Adoption	Productivity	Livelihoods
Publication type	Peer-reviewed journal article	51	42	10	12
	Book chapter	1	1	1	1
	Conference proceeding	8	6	3	2
	Report	6	6	4	3
	Working paper	7	3	1	4
Year of publication	2016–2019	15	10	3	7
	2011–2015	21	19	7	6
	2001–2010	28	24	6	8
	Before 2001	9	5	3	1
Agro-ecological zone	Mixed systems	53	41	12	15
	Agro-pastoral systems	10	9	2	2
	Pastoral systems	3	1	2	1
	Multiple systems	1	1	1	1
	Other	2	2		1
	(Blank)	4	4	2	2
Regions	Horn of Africa	12	11	2	3
	East Africa	23	19	5	6
	Central Africa	1	1		
	West Africa	7	5	2	1
	Southern Africa	5	5	2	1
	South Asia	6	4	1	2
	Southeast Asia	12	7	3	8
	East Asia	2	2	2	1
	Latin America	5	4	2	
Type of methods	Quantitative	45	40	14	13
	Qualitative	9	5		2
	Quantitative/qualitative	19	13	5	7
Duration of the experiment	>20 years	3	1	1	2
	11–20 years	4	4	1	1
	1–5 years	22	16	6	8
	6–10 years	8	6	3	5
	NA	36	31	8	6
**Total**		**73**	**58**	**19**	**22**

**Table 3:** Descriptive statistics of the results reported in the included studies, by level (adoption, productivity and livelihoods) and type of feed intervention

Indicators	Planted forages	Agroforestry	Crop residues
Adoption			
*N* (total)	43	19	6
*N* (studies with usable data)	32	11	3
Adoption range	0–90%	8–87%	20–86%
Productivity			
*N* (total)	18	6	38
*N* (studies with usable data)	2	1	6
Productivity change range	10–30%	0%	7–61%
Livelihoods			
*N* (total)	18	7	1
Household income change range (*N*)	6–285% (5)	10–80% (3)	NA (0)
Gross margin change range (*N*)	58–519% (3)	239% (1)	NA (0)
Labour use change range (*N*)	−70 to −24% (5)	NA (0)	NA (0)

**Table 4:** Summary statistics for quality assessment

Quality levels	Quality of study methodology	Study methodology justification	Overall subjective quality
High	17	31	17
High-medium	0	6	12
Medium	24	14	19
Medium-low	0	8	10
Low	32	14	15
Total	73	73	73

Box 1 Feed interventions and livestock production systems A livestock feed intervention aims at changing practices to provide more or better feed, increasing livestock productivity. The feed interventions considered in this scoping review are of three types:

1. Improved grasses and legumes—ruminant animals naturally consume grass, forbs or shrubby vegetation. Natural pastures, while of moderate quality, are an important feed source in low-income countries. ‘Improved’ species include naturally high-yielding tropical grasses such as *Brachiaria* or *Pennisetum* species as well as high-quality legume species such as vetches or *Desmodium*. If well managed, introduced grasses can greatly increase feed yields, while introduced high-protein legumes can complement basal feeds such as straws and natural pasture.
2. Multipurpose trees—trees are important in mixed crop–livestock systems, providing multiple benefits to small-scale farmers, including livestock feed. Various (mainly leguminous) trees have been popularized in tropical regions over recent decades. If well managed, these can provide a highly digestible and high-protein livestock feed and are reasonably resilient to dry spells.
3. Increasing the intake and nutritive value of crop residues—crop residues make up a large part of ruminant livestock feed across the tropics, particularly in semi-intensive crop–livestock systems. Residues include straws of cereals such as wheat, barley and rice; stems or leaves of cereals such as maize and sorghum; and legume straws or haulms. Crop residues are generally characterized by low nutrient density, especially cereal straws. Methods such as physical and chemical treatments as well as the selection of superior varieties have been developed to improve nutrient availability.

Other feed interventions include preserving fresh feed, filling seasonal gaps and feeding with high-quality supplements.

To describe global livestock production, various categorization approaches have been suggested. The most widely known was developed by Seré and Steinfeld^25^ and further operationalized by Thornton et al.^26^. It is based on major climate and land-use categories for which data are available globally and that determine the livestock feed base: permanent grasslands support pastoral systems, which often involve seasonal movement of livestock and are focused on ruminants; land areas that are used for both cropping and grazing are home to agro-pastoral and smallholder mixed crop–livestock systems, with or without irrigation; and landless systems rely solely on purchased feeds and are typically dominated by monogastric species, such as chickens and pigs, although landless urban dairy systems and feedlot beef systems also exist. More recently, researchers have suggested expanding this categorization to also consider the potential for the intensification of production, because of its considerable implications for development, using the evolution from subsistence to market orientation as a proxy^27,28^.

Box 2 Summary of methods

1. A comprehensive literature search for CAB Abstracts was created (see the search strategy at https://osf.io/5ec9k/).
2. The CAB Abstracts search strategy was translated to 22 additional bibliographic databases and grey literature sources (see the list of sources searched at https://osf.io/kghtc/).
3. To ensure accountability and reduce bias, a protocol was registered before data collection at https://osf.io/6ywht/.
4. After all bibliographic databases and sources were searched, the results were combined, duplicates were removed and 22,981 unique records were identified.
5. Using inclusion and exclusion criteria specified in the protocol, the records were screened blindly by two authors at each step, with a third author as a tie-breaker. The screening comprised three steps:
  - A machine learning process that used metadata to identify populations, geographies, interventions and outcomes of interest was applied. This process generated Excel files that could be quickly sorted and screened; 12,195 records were excluded, leaving 10,786 records.
  - The titles and abstracts of the remaining records were screened; 10,243 records were excluded, leaving 543 records.
  - The full texts of the remaining records were retrieved and screened; 470 records were excluded, leaving 73 records that were included in this scoping review.

After the full-text screening, 73 papers were identified that met our PRISMA-P a priori inclusion criteria and were included in the analysis, as shown in Tables 1, 2 and 3. Data were extracted to document all themes of interest including types of feed interventions, type of analysis, outcome variables and reported effects.

The inclusion criteria for this scoping review were:

1. The study focus includes a population of small-scale and agro-pastoral keepers of large and small ruminants.
2. The study is primary empirical research.
3. The explicit population focus is small-scale and agro-pastoral ruminant livestock keepers.
4. The study describes the adoption of ‘improved feed options’ and/or their effect on productivity, livelihoods or both.
5. The study area or focus includes target populations in LMICs.
6. The study is in English, French, Spanish or German.

High proportion of excluded articles: the highly inclusive search process returned many false positives. Thousands of irrelevant records were excluded at the title and abstract screening phase. Of the 470 articles excluded at the full-text screening, 257 did not include analysis of farmers’ adoption and/or the effect of the feed intervention on livestock productivity or livelihoods, and instead addressed feed trials and experiments.

Quality control: a subjective quality assessment was employed to categorize each study. Three criteria were used: the quality of the study methodology, the justification of the study methodology and an overall subjective quality. Table 4 summarizes these results.

Decision to use the scoping review methodology: scoping reviews are useful for incorporating a heterogeneous range of study designs typically found in agriculture. Unlike traditional narrative reviews, scoping reviews aim to consider all evidence on a topic and to reduce author, publication, confirmation and other forms of bias. Other evidence syntheses, such as meta-analyses (which aggregate quantitative results) and systematic reviews (which rely on homogeneous study methodologies and address intervention and outcome scenarios), did not fit the exploratory nature of this scoping review and the available evidence base.

## Results

As shown in Tables 1 and 2, there has been increased attention to and documentation of feed intervention adoption processes over time: in our final set of 73 included papers, 9 were published before the year 2000, 28 between 2000 and 2010, and 36 between 2011 and 2019. This increase in the adoption literature complements the more common technically oriented papers (mainly feeding trials) that dominated the earlier literature.

In terms of geographical representation, almost half of the papers (35) analysed sites in East Africa or in the Horn of Africa. Southeast Asia was the second most represented region (12 papers). The dominance of papers from these two regions is probably due to relevant development and research projects that have been implemented there, including those by centres of CGIAR. The two regions are also over-represented in the scoping review because unlike South America, they are dominated by small-scale livestock systems, which was an inclusion criterion. The relatively few papers from West Africa, despite our inclusion of papers in French, may be explained by the dominance of pastoral systems in West Africa, where feed interventions are more challenging. Indeed, in such systems (see Box 1 for a description of livestock systems) where livestock are kept with limited external inputs and husbandry is based on transhumance and extensive grazing, there are limited incentives for producers to invest in an improved technology that requires more resources (such as labour and finance) with uncertain effects on productivity and livelihoods, given the climatic and market risks.

Most of the papers (43) used quantitative analysis only, 19 used mixed methods and 9 papers followed qualitative research methods. The duration of the experiment was measured as the time lapse between the year the feed technology was introduced and the year the analysis was conducted. This was reported in slightly more than half of the reviewed papers (36 papers). Twenty-two papers reported a duration between 1 and 5 years, eight papers reported a duration between 6 and 10 years, and the remaining seven papers analysed cases longer than 11 years. The effects of technology adoption on ruminant productivity and the resulting impacts on households’ livelihoods can be observed only after some time, given that years are needed for the feed intervention to be implemented (for example, a new forage to grow and be harvested and fed) and for livestock productivity to increase, especially in the case of large ruminants.

In terms of agro-ecological zones and livestock systems (Box 1), 53 papers covered mixed systems, and only 13 analysed pastoral and agro-pastoral systems, an expected result given the difficulty of introducing new feed interventions in these systems, as explained above. Livestock exclosures (areas of land that are protected from grazing animals) and fodder banks (areas reserved to grow fodder to be used during the dry season, usually trees, shrubs and fodder legumes) were the main interventions in pastoral/agro-pastoral systems.

### Types of feed interventions and impact pathway

The majority of papers (53) dealt with planted fodder, while agroforestry was the topic of 26 papers and crop residues 7 papers (Table 1). Most papers assessed one type of feed intervention (30 papers on planted fodder only, and 13 and 4 for agroforestry and crop residues, respectively). There seemed to be a mismatch between the research effort on feed intervention types and the importance of the technologies in overall livestock feeding. Crop residues constitute a large part of feed resources in small-scale ruminant systems, and they have great potential to be even more productive. Yet less than one in ten articles dealt with crop residues. Crop residues have no dedicated discipline and low visibility in terms of impacts despite their ubiquity across tropical livestock systems. After harvest, crop residues are bulky and may be complex to manage, in terms of storage, labour demand and seasonal availability. In contrast, about a third of the papers covered agroforestry interventions, a relatively less prominent feed option in these systems. This may be explained by the fact that agroforestry is used for feed as well as soil fertility, among other purposes. There could also be greater charisma associated with trees than with straw.

This scoping review analysed the impact pathway between adoption, livestock productivity and household livelihoods (Table 2), considering three main outcomes. The first was about the uptake, or adoption, of feed technologies (58 papers). We considered studies of livestock keepers using new feed interventions as part of their usual management practices independent of incentives-based research or development projects. Second, we were interested in studies on livestock productivity increases including milk production, weight gain, better body condition or herd growth that resulted from a feed intervention (19 papers). The final outcome of interest was household livelihood indicators associated with the uptake of a new feed option and consequently improvements in livestock productivity (22 papers). Such livelihood changes included increased income from livestock and reduced workload. Of the 73 papers, only 6 analysed the entire pathway, reporting evidence of adoption, the effect on productivity and consequent livelihood changes.

Adoption is the first step along the livelihood pathway, and it was anticipated to feature in most papers, given that it is relatively easy to measure either as a yes/no decision or as the extent of adoption. We found 15 papers that did not explicitly report or analyse that first step but only reported a change in productivity and/or livelihood indicators among adopters. More frequently, papers reported livelihood changes compared to productivity changes. This may be explained by a focus on development goals, such as producers’ livelihoods, and by the technical challenges of measuring livestock productivity. Livelihood indicators such as income and diet are relatively well established, and tools are available to systematically report them. In contrast, livestock productivity indicators vary across species (such as milk yield, weight gain, herd size and fertility indicators), require demanding measurement protocols and are calculated using different periods (such as lactation, reproductive cycle, season and year), making comparisons difficult.

Across the three outcome sets, there were no specific differences in types of publication or years of publication. Studies in mixed systems dominated across all outcomes, with few papers covering agro-pastoral or pastoral systems. For regions, papers from East Africa were most common for adoption studies, while for livelihood outcomes, there was a relatively large number of papers from Southeast Asia (8 out of 22 papers), possibly driven by research in development projects implemented in that region. In terms of research methods, the general observation that studies were mainly quantitative applied to all three outcomes. There were more mixed-methods approaches for adoption studies, possibly reflecting the importance of not only analysing the decision-making processes quantitatively but also assessing the ‘why’ and the ‘how’ that are better captured using qualitative approaches. There was no qualitative study measuring productivity indicators.

### Analysis of the results reported by the studies

The outcome indicator results reported in the studies are shown in Table 3. Although 43 studies reported the adoption of forages, only 32 included data that could be used to estimate adoption. The same pattern applied to the adoption of agroforestry practices and crop residues. Analysing the results as reported in these studies, we found that the ranges of livestock keepers adopting the technology varied widely, from 0 to 90% for forages, 8 to 87% for agroforestry and 20 to 86% for crop residues.

Productivity indicators included increase in milk yields, animal weight gain, improved body condition and growth in flock/herd size. The number of papers with sufficient data was very low, with only nine papers across the three feed interventions. Changes in productivity ranged from 7 to 61%, with only one paper reporting productivity change related to crop residues.

Finally, for livelihood indicators, the scoping review identified 22 papers with sufficient data across the three feed types, with 14 papers quantifying the impact. Household income change (8 papers) ranged from 6 to 285%, gross margins (3 papers) increased by 58 to 519% and labour or workload change (5 papers) from −24 to −70%.

### Drivers of adoption

To better understand the reported changes, 25 papers were identified that explicitly examined the reasons for adoption. These were further examined for underlying drivers or constraints to adoption. Of the adoption drivers, the following were mentioned most often. Farmer experience or level of education was mentioned in ten papers; these variables are commonly collected as part of the household characterization in adoption studies and tend to be associated with higher rates of adoption. Expected increased productivity or income from the livestock enterprise was mentioned in eight papers. This is of course among the primary reasons to promote improved feed technologies, so this factor is expected to be prominent. However, most of the papers did not indicate it. Eight papers mentioned access to extension or training. Many feed technologies require considerable technical skill to be successful or effective. These are often described as ‘knowledge-intensive’ technologies, such as forage seeds that require treatment or scoring to germinate and then need to be grown from seedlings. Extension and training may therefore be important to facilitate their successful implementation. Seven papers mentioned labour availability. Most improved feed technologies require the use of additional and regular labour, such as cutting and carrying planted forages to confined ruminants. Family labour may be supplemented in some seasons by casual wage labour or even full-time labour in more market-oriented enterprises. Six papers mentioned good market access; again, this factor is generally associated with higher rates of adoption and may be associated with higher livestock product prices or easier access to feed technologies such as germplasm. Other contributing factors, in descending order, were access to credit or off-farm income, market orientation of the enterprise, group membership or social pressure, and land scarcity. Only two studies indicated soil improvement as an adoption objective, although this is one of the main reasons that nitrogen-fixing leguminous forages are promoted.

Of the factors that were indicated as constraining the adoption of improved feed technologies, the following were mentioned most frequently. Increased labour requirement was mentioned in six papers; just as labour availability was indicated as an important driver of adoption, the labour requirement can be a constraining factor when that labour is not easily available. Little perception of net benefit was mentioned in four papers. Feeds are an intermediate output towards livestock production, and the final benefit may not be easily perceived immediately, particularly for fattening enterprises where nutritional benefits accrue over longer periods. Four papers mentioned difficult access to the technology or inputs. For some forage species, there may be limited systematic supply of seeds or planting material, and this is often a limit to sustained use after the withdrawal of project support. Many LMICs lack functioning forage seed systems. Four papers mentioned the complexity of the technology; as indicated, some feed technologies may require specific techniques, the training in which may not be available. Finally, competition with other land uses was mentioned in four papers. In land-scarce settings, priority may be given to food crops or to short-term cash crops such as seasonal vegetables, since these may represent a more profitable use of land. Likewise, some alternative land uses may be affected by subsidies and price control and may influence the relative returns from some feeding options.

### Quality assessment

The research quality assessment was conducted using three indicators for all 73 papers (Table 4). In terms of study methodology, 17 papers scored high, and almost half of the papers (32) scored low. The quality assessment on the justification of the study methodology was slightly better, with 31 papers being scored high. The scores for the overall quality were relatively evenly distributed, with 17 papers having the highest scores and 15 the lowest ones. Overall, the quality of the papers was judged to be average to low. Both the number and quality of studies that were included in this analysis are rather disappointing, given the role that improved feed options can and should play in enhancing livestock productivity and household livelihoods.

## Discussion

First, it is worth noting that the scoping review identified very few studies that answer our research question on the comparative impacts of various ruminant feed interventions on the livelihoods of livestock keepers. Indeed, the exercise yielded only 73 papers from a starting population of 22,981. We found many papers that studied the technical aspects of feed supply for ruminant livestock but were excluded because they did not assess the interventions’ uptake by or usefulness to farmers. This points to a strong bias among the scientific community towards understanding the technical intricacies of ruminant feeding without paying sufficient attention to how such technologies fit into general farming practices or farmer objectives. Additionally, a number of studies were dropped as per the exclusion criteria because they focused on large-scale livestock production. Several studies from Latin America fell in this category, which is consistent with the fact that farms in Latin America and the Caribbean are generally larger than farms in other regions, including sub-Saharan Africa and Asia^15^.

Second, among the few papers included in the final analysis, the majority only analysed the adoption of feed interventions, and only six studies additionally documented the productivity and livelihood impact pathways of the feed interventions. The funding of research to generate rigorous and relevant evidence of feed innovation outcomes and impacts has been restricted mainly to those development projects introducing such innovations. However, such development-project-linked research may not be able to analyse the whole pathway from adoption to animal and household impact given the limited lifespans of such projects, particularly since productivity gains may only translate to sustained herd growth over time, and project termination may lead to the withdrawal of needed farmer support services.

Third, the literature was found to be skewed towards forages and agroforestry, yet crop residues are among the largest sources of basal feed for ruminant livestock across tropical regions (in addition to natural pasture). This apparent mismatch may result from the fact that forages and multipurpose trees can offer a step change in productivity, compared with the more incremental productivity benefits from the improved use of crop residues. Forages and trees also have a disciplinary home, with whole research institutes devoted to their study. Crop residues are generally seen as by-products of human food production despite the fact that the market value of straw can in some cases approach that of grain for human food^16^. The focus on human food production by cereal breeding research institutes leaves the residues as an ‘orphan crop’. Our scoping review points to the need to focus more research effort on improving crop residue yields and quality characteristics such as through orienting crop breeding towards improving the feed quality of underutilized residues^17^. Practices involving improving crop residue quality and yield may have a strong likelihood of adoption, since few changes to farming practices are required in contrast to planted forages and forage trees.

Fourth, no clear conclusion emerged from comparing the effects of various feed-oriented interventions. Indeed, the ranges of change indicators presented are so large that meaningful comparisons are difficult. Several factors seem to have contributed to this. First, the intervention categories (that is, planted forages, agroforestry and crop residues) contain a wide variety of individual interventions with very different potentials for inducing change. For instance, introducing a new forage crop into a system without any prior forage cultivation can yield substantial improvements in productivity^18^ compared with the incremental effects of introducing a new variety of an established forage species^19^. Second, the approaches to determine intervention impacts differ considerably between studies. Where a development project is focused on development impact (for instance, by creating an enabling environment for farmers to adopt or by targeting mainly high-potential beneficiaries), outcomes are likely to be greater than in an independent study aiming to determine how farmers benefit from a variety of interventions. An example of the former is presented by Roothaert and Kerridge^20^, reporting a gross margin increase of 239% among project participants, whereas a study on various fodder shrubs in central Kenya was able to detect an income improvement of only 10% (ref. ^21^). Third, the time horizon considered by the reviewed studies varies greatly (Table 2). Most studies report changes only for the entire study period rather than average annual changes. Also, the rate of change brought about by feed interventions might not be constant. It is probable that a single intervention would generate change along an S-curve with only little evidence of change initially, followed by a period of considerable change, after which the rate would decrease. The reported rates may refer to very different periods within the change processes. Finally, the success of land-based interventions, such as those targeting feeds, is generally very site-specific, depending on biophysical features (such as rainfall or temperature) as well as on social characteristics (such as land prices or market access). The reviewed studies cover a wide range of such features and characteristics, from densely populated and humid Philippines and Vietnam^22^ to mountainous Nepal^23^, showing increases in household income of 285% and 11%, respectively.

Finally, this scoping review has identified various factors driving or constraining the adoption of feed interventions, which can be grouped into three broad and inter-related categories. The first category refers to managing a sometimes-challenging technology, requiring certain skills on the part of the farmer as well as access to the technology, to extension and to training in its use. Second, the benefits of using a technology and its alignment with the farmer’s objectives must be perceived and valued by the farmer for adoption to occur. This is often an issue because feed is an intermediate technology in the livestock value chain, and the link between better feeding and financial benefits may not be easily perceived. Furthermore, livestock may be kept for a range of reasons other than the production of milk and meat, and the farmers’ primary objective may not be immediately obvious to well-meaning development agents. Third, the availability of the key resources of land and labour, and the trade-offs between them that the feed technology may impose, will limit or facilitate adoption, with adequate availability of both (particularly labour) generally having a positive effect. The trade-offs in certain contexts may mean that farmers can derive greater benefit from allocating land and labour to non-livestock activities, and this needs careful consideration when considering feed interventions.

The consideration of these adoption drivers and constraints is helpful for considering future approaches to enhancing livestock feed supply among poor livestock keepers. Too often, technologies have been promoted without systematically considering barriers to their uptake, whether target farmers have sufficient resources (both financial and human) to successfully implement them and whether the technologies make economic sense given the market conditions and the competing opportunities for the use of land and labour in target communities. Box 3 presents recommendations for researchers and development practitioners.

Box 3 Recommendations This scoping review has shown that besides technical feed efficiency characteristics, various other factors enhance or constrain the adoption of improved feeds. On the basis of our analysis, we recommend the following:

- For ‘knowledge-intensive’ technologies, the capacity of local livestock keepers and the strength of the extension advice environment to support ongoing implementation should be considered. If these are limited, some re-evaluation of the technology options or a parallel effort to enhance the necessary capacity among local livestock keepers is needed.
- In planning development efforts for livestock feeding, the focus needs to be on small-scale, semicommercial farmers who have both the resources and the incentive to make the investments needed for feed technologies to succeed. Livestock keepers whose primary objectives for keeping livestock are not to produce milk and meat for the market need a different kind of support and are much less likely to invest in new feed technologies.
- The resource requirements for livestock feed options need careful consideration. If other uses for land and labour are more lucrative, livestock keepers are unlikely to invest in new feed options. This requires the whole farming system to be considered, as well as how livestock fit into overall livelihood strategies. In addition, unlike food crops, forage seed systems are underdeveloped in many regions (especially sub-Saharan Africa), with unclear demand and limited supply from the private sector. Public–private partnerships and investment may be needed to develop these supply chains and can be linked to local seed-producing entrepreneurs and collective groups.
- Decision makers and development agents should consider these factors and constraints in deciding when and where to target investments promoting these technologies. The conditions that favour feed technology adoption go far beyond biophysical suitability, extending to the social, economic and knowledge domains.

## Methods

### Evidence synthesis methodology and protocol preregistration

This scoping review was conducted following the PRISMA-ScR (Preferred Reporting for Items for Scoping Reviews) checklist. A protocol was registered in Open Science Framework on 5 June 2019 at https://osf.io/6ywht/.

### Information sources, searches and citation management

A comprehensive search strategy was developed (by E.L. and E.E.) to identify all available research pertaining to the livelihoods of small-scale and agro-pastoral livestock keepers in LMICs in Africa, Asia and Latin America, in relation to the improvement of ruminant feed interventions. The search terms included variations of the key concepts in the research question: improvement or conservation of crops; small-scale producers or pastoralists; LMICs in Africa, Asia or Latin America; and innovation or adoption indicators. The comprehensiveness of the search strategy was ensured by including all known search-term synonyms and appropriate subject term searches, conducting a peer review of search strategy by expert librarians familiar with the discipline, and confirming the inclusion of eight seminal studies in the results set. See Supplementary Appendix A for the search strategy used for CAB Abstracts (accessed via the Clarivate Analytics platform).

On 5 June 2019, four bibliographic databases were searched. These included CAB Abstracts (Clarivate Analytics, 1910–present), Web of Science Core Collection (Clarivate Analytics, 1900–present), Scopus (Elsevier, 1970–present) and Dissertations and Theses Global (ProQuest, 1743–present). On the same day, 20 grey literature sources were searched, including Africa Theses and Dissertations, AgEcon Search, AGRIS, Campbell Collaboration, Cochrane Collaboration, Collaboration for Environmental Evidence, Commonwealth Scientific and Industrial Research Organization, Brazilian Agricultural Research Corporation (Embrapa), French Agricultural Research Centre for International Development, GARDIAN, International Fund for Agricultural Development, International Institute for Environment and Development, JPAL/ATAI Impact Evaluations, Overseas Development Institute, UK Department for Internal Development, United Nations Environment Programme, World Food Programme, World Health Organization, World Bank, and Cgspace.

The search results were deduplicated to remove citations identified in multiple databases. The titles, abstracts and keywords of all citations were exported as RIS files.

### Study selection and eligibility criteria

**Extended Data Fig. 1:**
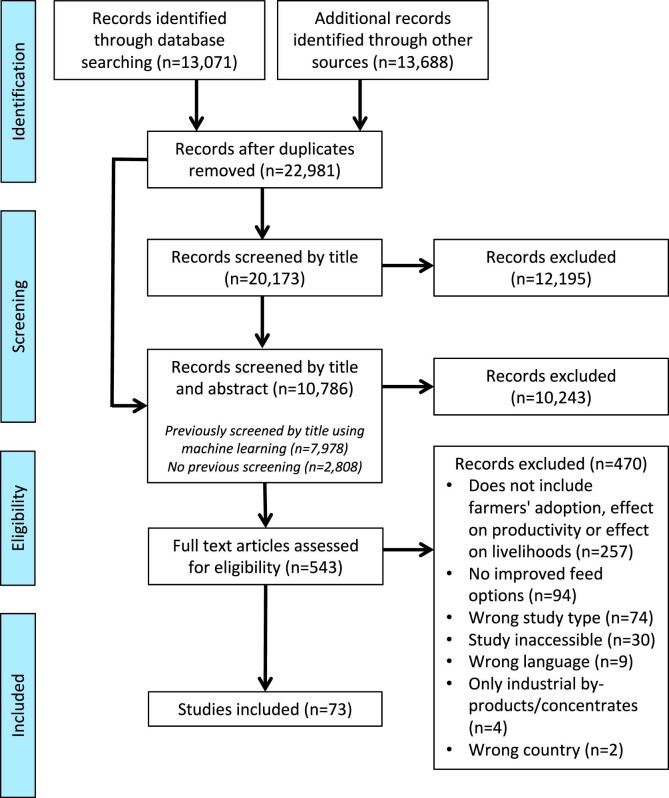
PRISMA flow diagram.

To accelerate the identification of potential articles for exclusion at the title stage, a machine learning process that used metadata to identify populations, geographies, interventions and outcomes of interest was applied. This process generated Excel files that could be quickly sorted and screened. The majority of the records (*N* = 20,173) were screened via this method, blindly, by author pairs (including I.B., E.J.R., E.E., E.L., R.L., A.D. and D.C.), and citations that both authors excluded did not progress to the title and abstract screening phase. The remaining studies were imported into Covidence, a systematic review screening software, and were screened blindly via title and abstract by two authors with a third as a tie-breaker (I.B., S.S., E.J.R., N.T., R.L., A.D. and D.C.). Studies that were not excluded by title and abstract screening were considered at the full-text level. These were screened blindly using the same inclusion and exclusion criteria, and conflicts were resolved by a third author as a tie-breaker (I.B., S.S., E.J.R., N.T., R.L., A.D. and D.C.). Individual reasons for exclusion were recorded at the full-text screening stage (Extended Data Fig. 1).

During all stages of screening (title, title and abstract, and full text), studies were excluded if they did not meet all of the following inclusion criteria: (1) the study focus includes a population of small-scale and agro-pastoral keepers of large and small ruminants; (2) the study is primary empirical research; (3) the explicit population focus is small-scale and agro-pastoral ruminant livestock keepers; (4) the study describes the adoption of ‘improved feed options’ and/or their effect on productivity, livelihoods or both; (5) the study area or focus includes target populations in LMICs; and (6) the study is in English, French, Spanish or German.

In addition, studies were excluded if they met one or more of the following exclusion criteria: (1) the study is a review or a case study; (2) the study does not include small-scale or agro-pastoral as the target population; (3) the study does not take place in LMICs in Latin America, Africa or Asia; (4) the study does not consider improved feed options (introduced by an external entity or the farmer’s own experimentation); (5) the study considers only industrial by-products and/or concentrates; (6) the study is in a language other than English, French, Spanish or German; and (7) the study considers only fish, pigs, poultry, camels, wild buffaloes, yaks, alpacas, guinea pigs (or cavies), bees, equines, rabbits or any wild animal.

### Data extraction and analysis

A data extraction template was created on the basis of Barrett et al.^24^ and adapted to the scoping review requirements. The data extracted included the author(s), year of publication, type of paper, study location, intervention type, comparator (if any), duration of the intervention, study population and methodology; the outcome measures differentiated by adoption, effects on livestock productivity and effects on livelihoods; and important results in terms of drivers of adoption and potential for scaling. The template was tested by I.B. and A.D. before being finalized. Google forms were used to extract the data by I.B., A.D., N.T., D.C., E.J.R., S.S. and R.L. Conflicts were resolved by a third author as a tie-breaker. The data analysis tables were created and data processed by N.T., I.B. and E.J.R. Data were extracted on 51 peer-reviewed journal articles, 8 papers published in conference proceedings, 7 working papers, 6 reports and 1 book chapter. The quality assessment was conducted on the 73 included papers using three criteria. The first one considered the quality of the study methodology (low versus high), the second assessed the justification of the methodology (low versus high) and the third criterion was an overall quality assessment with three levels (low, medium and high). Each paper was scored by two persons (D.C., A.D., I.B. and R.L.). The levels were first transformed into scores (high, 1; medium, 2; low, 3) and then averaged.

To better understand some of the underlying drivers of adoption of improved feed technologies, a subset of the final papers was selected for more detailed examination if they mentioned analysis of factors that either facilitated adoption or constrained adoption. Of the 73 papers in the full set, 25 met this criterion. Each of these papers was then re-examined by one researcher, and a set of adoption drivers and constraints was identified; the papers were scored on whether they mentioned each adoption driver or constraint. Twelve different adoption drivers emerged, such as increased productivity and good access to markets. Nine constraining factors were also indicated across this set of papers, such as low perceived benefit of the technology and competition with other land uses.

## Supplementary information

Supplementary Information Bibliography of the final list of studies, data extraction template and protocol, including all final search strategies.

## Data Availability

The data that support the findings of this study are available from the corresponding author upon request.
