# Supplementary material for: A scoping review of feed interventions and livelihoods of small-scale livestock keepers
Source: Nat Plants. 2020 Oct 12;6(10):1242–9. doi: 10.1038/s41477-020-00786-w (PMC7553850; doi:10.1038/s41477-020-00786-w)
Supplement: Supplementary file 1 — Bibliography of the final list of studies, data extraction template and protocol, including all final search strategies. [file 41477_2020_786_MOESM1_ESM.pdf]

natureresearch
https://doi.org/10.1038/s41477-020-00786-w

Supplementary information

A scoping review of feed interventions and livelihoods of small-scale livestock keepers

In the format provided by the
authors and unedited

Bibliography of the final list of studies

1. Abebe, A., Hagos, A., Alebachew, H., Faji, M., Abebe, A., Hagos, A., Alebachew, H., & Faji, M. (2018). Determinants of adoption of improved forages in selected districts of Benishangul-Gumuz, Western Ethiopia. Tropical Grasslands-Forrajes Tropicales, 6(2), 104–110. https://doi.org/10.17138/tgft(6)104-110
2. Adie, A., Duncan, A. J., & Ergano, K. (2010). Participatory evaluation of planted forages in Ada'a, Miesso and Alamata woredas of Ethiopia [Report]. ILRI. https://cgspace.cgiar.org/handle/10568/3014
3. Ashley, K., Wilson, S., Young, J., Chan, H., Vitou, S., Suon, S., Windsor, P., & Bush, R. (2018). Drivers, challenges and opportunities of forage technology adoption by smallholder cattle households in Cambodia. Tropical Animal Health and Production, 50(1), 63–73. https://doi.org/10.1007/s11250-017-1400-y
4. Ashley, K., Young, J. R., Kea, P., Suon, S., Windsor, P. A., & Bush, R. D. (2018). Socioeconomic impact of forage-technology adoption by smallholder cattle farmers in Cambodia. Animal Production Science, 58(2), 393–402. https://doi.org/10.1071/AN16164
5. Assefa, H., Egziabher, T. G, & Tegegne, A. (2015). Agricultural knowledge management: The case of cattle feed quality improvement in Bure district west Gojjam, Ethiopia. Journal of Agricultural Extension and Rural Development. https://dx.doi.org/10.5897/JAERD2014. 0641
6. Atuhaire, A. M., Mugerwa, S., Okello, S., Lapenga, K. O., Kabi, F., & Kabirizi, J. M. (2014). Prioritization of Crop Residues for Improving Productivity on Smallholder Dairy Farming Households in the Lake Victoria Crescent, Uganda. Open Journal of Animal Sciences, 04(02). https://doi.org/10.4236/ojas.2014.42014
7. Ayele, Z. (2003). Community-based forage development program: The experiences of Farm Africa Goat Project in Ethiopia. Tropical Grasslands, 37(4).
8. Baltenweck, I., Staal, S. J., Ibrahim, M. N. M., Herrero, M., Holmann, F., Jabbar, M., Manyong, V., Patil, B. R., Thornton, P. K., Williams, T., Waithaka, M., & de Wolff, T. (2003). Crop-Livestock Intensification and Interactions Across Three Continents: Main Report. ILRI, CIAT, IITA, University of Peradeniya, BAIF, Nairobi, Kenya.
9. Bannister, M. E., & Nair, P. K. R. (1990). Alley cropping as a sustainable agricultural technology for the hillsides of Haiti: Experience of an agroforestry outreach project. American Journal of Alternative Agriculture, 5(2), 51–59. https://doi.org/10.1017/S0889189300003283
10. Bardhan, D., Dabas, Y. P. S., & Kumar, A. (2005). Impact assessment of dissemination of improved livestock practices. Livestock International, 9(7), 15–19.
11. Beshir, H. (2014). Factors Affecting the Adoption and Intensity of Use of Improved Forages in North East Highlands of Ethiopia. American Journal of Experimental Agriculture, 4(1). https://doi.org/10.9734/ajea/2014/5481
12. Birhanu, M. Y., Girma, A., & Puskur, R. (2017). Determinants of Success and Intensity of Livestock Feed Technologies Use in Ethiopia: Evidence from a Positive Deviance Perspective. Technological Forecasting and Social Change 115(March 2019), 15–25.
13. Bosma, R. H., Roothaert, R., &p Ibrahim.(2001). Economic and Social Benefits of New Forage Technologies in East Kalimantan, Indonesia. Vol. CIAT Worki. CIAT, Los Banos, Phillipines.
14. Bosma, R., Roothaert, R., Asis, P., Saguinhon, J., Binh, L., & Yen, VH. (2003). Financial and social benefits of new forage technologies in Mindanao, Philippines and Tuyen Quang, Vietnam. http://agris.fao.org/agris-search/search.do?recordID=PH2003001044
15. Derseh, M. B., Duncan, A. J., Mekonnen, K., Adie, A., Khan, N. A., & Thorne, P. (2016). The role of irrigated fodder production to supplement the diet of fattening sheep by smallholders in

southern Ethiopia. Tropical and Subtropical Agroecosystems, 19(3). http://www.revista.ccba.uady.mx/ojs/index.php/TSA/article/view/2215

16. Ekumankama, O. O. (1999). Managing Natural Pasture for Small Ruminants: The Case of Alley Farming in Ikwuano Area of Abia State. Nigerian Journal of Animal Production 26, 120–124.
17. Floyd C.N., Harding, A.H., Paddle, K.C. Rasali, D.P., Subedi, K.D. & Subedi, P.P. (1999). The adoption and associated impact of technologies in the western hills of Nepal. ODI Agricultural Research & Extension Network. Network Paper No 90. https://www.odi.org/sites/odi.org.uk/files/odi-assets/publications-opinion-files/5142.pdf
18. Franzel, S, Arimi, H., & Muriithi, F. M. (2002). Calliandra calothyrsus: Assessing the early stages of adoption of a fodder shrub in the highlands of central Kenya. In In World Agroforestry | Transforming Lives and Landscapes with Trees (pp. 125–144). CABI Publishing. http://www.worldagroforestry.org/publication/calliandra-calothyrsus-assessing-early-stages-adoption-fodder-shrub-highlands-central
19. Franzel, S., Wambugu, C., Tuwei, P., & Karanja, G. (2003). The Adoption and Scaling up of the Use of Fodder Shrubs in Central Kenya. Tropical Grasslands 37(4), 239–250.
20. Franzel, Steve, Wambugu, C., & Tuwei, P. (2003). The adoption and dissemination of fodder shrubs in central Kenya (No. 131). https://www.odi.org/sites/odi.org.uk/files/odi-assets/publications-opinion-files/5190.pdf
21. Fujisaka, S., Rika, I. K., Ibrahim, T. ,& An, L. Van. (2000). Forage Tree Adoption and Use in Asia. In: Horne, P., Hacker, J. B., Kerridge, J. B., and Peter, C. (Eds.). Working with farmers: the key to adoption of forage technologies. Proceedings of an International Workshop held in Cagayan de Oro City, Mindanao, Philippines from 12-15 October 1999, pp. 243–253. ACIAR, Canberra, Australia.
22. Gabb, S., Bell, L., Basuno, E., Prestwidge, D., Prior, J., & Guppy, C. (2017). Whole farm impacts of forage legumes in smallholder crop-livestock systems. Agronomy Australia Proceedings, 4. http://agronomyaustraliaproceedings.org/index.php/2017
23. Gabunada Jr., F., Heriyanto, Phengsavanh, P., Phimphachanhvongsod, V., Truong Thanh, K., Nacalaban, W., Asis, P., Vu Thi Hai, Y., Tugiman, Ibrahim, & Stur, W. W. (2000). Integration of adapted forages on farms in Southeast Asia - experiences from the forages for Smallholders Project. In Working with farmers: the key to adoption of forage technologies. Proceedings of an International Workshop held in Cagayan de Oro City, Mindanao, Philippines from 12-15 October 1999.
24. Gebremedhin, B., Ahmed, M. M., & Ehui, S. K. (2003). Determinants of Adoption of Improved Forage Technologies in Crop-Livestock Mixed Systems: Evidence from the Highlands of Ethiopia. Tropical Grasslands 37(4), 262–273.
25. Hamer, A. G., Franzel, S., & Mounkoro, B. (2007). Using farmers' criteria to assess profitability of fodder shrubs in the desert margins of West Africa. Land Degradation and Development, 18(6). https://doi.org/10.1002/ldr.805
26. Hau, D. K., Panjaitan, T., Nulik, J., Dahlanuddin, & van de Fliert, E. (2014). Barriers to and opportunities for the use of forage tree legumes in smallholder cattle fattening systems in Eastern Indonesia. Tropical Grasslands-Forrajes Tropicales, 2(1). https://doi.org/10.17138/tgft(2)79-81
27. Hove, L., Franzel, S. & Moyo, P. S. (2003). Farmer Experiences in the Production and Utilisation of Fodder Trees in Zimbabwe: Constraints and Opportunities for Increased Adoption. Tropical Grasslands 37(4), 279–283.
28. Jera, R., & Ajayi, O. C. (2008). Logistic Modelling of Smallholder Livestock Farmers' Adoption of Tree-Based Fodder Technology in Zimbabwe. Agrekon 47(3), 379–392.

29. Jorge, M., Lukuyu, B., Marita, C., Mwangi, D., Kinuthia, E., Baltenweck, I., & Poole, E. J. (2014). Assessing the uptake and disease impact of Napier grass in Kenya. https://cgspace.cgiar.org/handle/10568/51336
30. Kaufmann, R. R. von, & Mohamed-Saleem, M. A. (1989). Interactions between agronomy and economics in forage legume research. ILCA Bulletin. https://cgspace.cgiar.org/handle/10568/4569
31. Kebebe, E.G. (2017). Household nutrition and income impacts of using dairy technologies in mixed crop–livestock production systems. Aust J Agric Resour Econ, 61: 626-644. doi:10.1111/1467-8489.12223
32. Kebebe, E. G., Oosting, S. J., Baltenweck, I., & Duncan, A. J. (2017). Characterisation of adopters and non-adopters of dairy technologies in Ethiopia and Kenya. Tropical Animal Health and Production, 49(4), 681–690. https://doi.org/10.1007/s11250-017-1241-8
33. Khan, Ho Le Phi, Jeff Corfield, Nguyen Xuan Ba and David Parsons. (2015) The best-bet participatory approach and its impact on smallholder livelihood Sustainable and profitable crop and livestock systems in south-central coastal Vietnam Proceedings of the final workshop held in Quy Nhon, Vietnam, 5–6 March 2013 Editors: Surender Mann, Mary C. Webb and Richard W. Bell; ACIAR PROCEEDINGS 143 pp208-218
34. Khanh, T., Anh, N., Stür, W., Tiemann, T., & Duncan, A. (2011). Process of up-scaling fodder development: Viet Nam case. https://cgspace.cgiar.org/handle/10568/4743
35. Kiptot, E., Lukuyu, B., Franzel, S., & Place, F. (2011). The farmer trainers approach in technology dissemination in Uganda: Farmer trainers’ and trainees’ perspectives.
36. Kristjanson, P., Tarawali, S., Okike, I., Singh, B., Thornton, P., Manyong, V., Kruska, R., & Hoogenboom, G. (2002). Genetically improved dual-purpose cowpea. Assessment of adoption and impact in the dry savannah of West Africa. https://cgspace.cgiar.org/handle/10568/508
37. Kumar, M., Singh, R. P., & Misra, A. K. (2015). Adoption level of green fodder production practices and constraints faced by the farmers of Rajasthan. Range Management and Agroforestry, 36(2).
38. Lapar, M. L. A., & Ehui, S. K. (2004). Factors affecting adoption of dual-purpose forages in the Philippine uplands. Agricultural Systems, 81(2). https://doi.org/10.1016/j.agsy.2003.09.003
39. LiMin, H., Lian, Y., Michalk, D., & Jianping, W. (2008). Effect of using cultivated forages on herder’s income in three types grassland ecological zones. In Multifunctional Grasslands in a Changing World (p. 2).
40. Mapiye, C., Foti, R., Chikumba, N., Poshiwa, X., Mwale, M., Chivuraise, C., & Mupangwa, J. F. (2006). Constraints to Adoption of Forage and Browse Legumes by Smallholder Dairy Farmers in Zimbabwe. Livestock Research for Rural Development 18(12), 1–11.
41. Mekoya, A., Oosting, S. J., Fernandez-Rivera, S., & Van der Zijpp, A. J. (2008). Farmers’ perceptions about exotic multipurpose fodder trees and constraints to their adoption. Agroforestry Systems, 73(2), 141–153. https://doi.org/10.1007/s10457-007-9102-5
42. Mganga, K. Z., Musimba, N. K. R., Nyariki, D. M., Nyangito, M. M., & Mwang’ombe, A. W. (2015). The choice of grass species to combat desertification in semi-arid Kenyan rangelands is greatly influenced by their forage value for livestock. Grass and Forage Science, 70(1). https://doi.org/10.1111/gfs.12089
43. Millar, J., & Connell, J. (2010). Strategies for scaling out impacts from agricultural systems change: The case of forages and livestock production in Laos. Agriculture and Human Values, 27(2). https://doi.org/10.1007/s10460-009-9194-9
44. Mudzengi, C. P., Taderera, L. M., Tigere, A., Kapembeza, C. S., Moyana, S., Zimondi, M., Derembwe, E. T., & Dahwa, E. (2014). Adoption of urea treatment of maize stover technology

for dry season supplementation of cattle in Wedza, Zimbabwe. Livestock Research for Rural Development, 26(9).

45. Mugerwa, S., Kabirizi, J.M., Njarui, D., Mpairwe, D. (2012). Utilization of introduced forages by smallholder dairy farmers in Uganda. International Journal of Biosciences, 1, 36–45.
46. Muhr, L., Tarawali, S. A., Peters, M., & Schultze-Kraft, R. (2001). Acceptability of forage legumes for improved fallows - First experiences of agro-pastoralists in subhumid Southwest Nigeria. Experimental Agriculture, 37(4). https://doi.org/10.1017/s0014479701000436
47. Mureithi, J. G., Njunie, M. N., Muinga, R. W., Ali, R., Thorpe, W., & Mwatate, C. D. (1998). Adoption of planted forages by smallholder dairy farmers in coastal lowland Kenya. Tropical Grasslands, 32(4).
48. Mureithi, S. M., Verdoodt, A., Njoka, J. T., Gachene, C. K. K., & van Ranst, E. (2016). Benefits Derived from Rehabilitating a Degraded Semi-Arid Rangeland in Communal Enclosures, Kenya. Land Degradation and Development, 27(8). https://doi.org/10.1002/ldr.2341
49. Mutambara, J., Dube, I. v., & Mvumi, B. M. (2012). Agroforestry technologies involving fodder production and implication on livelihood of smallholder livestock farmers in Zimbabwe. A case study of goromonzi district. Livestock Research for Rural Development, 24(11).
50. Mwangi, D. M., & Wambugu, C. (2003). Adoption of forage legumes: The case of Desmodium intortum and Calliandra calothyrsus in central Kenya. Tropical Grasslands, 37(4), 227-238.
51. Njarui, D. M. G., Gatheru, M., Gichangi, E. M., Nyambati, E. M., Ondiko, C. N., & Ndungu-Magiroi, K. W. (2017). Determinants of forage adoption and production niches among smallholder farmers in Kenya. African Journal of Range and Forage Science, 34(3). https://doi.org/10.2989/10220119.2017.1387814
52. Omollo, E. O., Wasonga, O. V., Elhadi, M. Y., & Mnene, W. N. (2018). Determinants of pastoral and agro-pastoral households' participation in fodder production in Makueni and Kajiado Counties, Kenya. Pastoralism, 8(1). https://doi.org/10.1186/s13570-018-0113-9
53. Pandit, B. H., Nuberg, I., Shrestha, K. K., Cedamon, E., Amatya, S. M., Dhakal, B., & Neupane, R. P. (2019). Impacts of Market-Oriented Agroforestry on Farm Income and Food Security: Insights from Kavre and Lamjung Districts of Nepal. Agroforestry Systems 93(4), 1593–1604.
54. Paul, B. K., Muhimuzi, F. L., Bacigale, S. B., Wimba, B. M. M., Chiuri, W. L., Amzati, G. S., & Maass, B. L. (2016). Towards an assessment of on-farm niches for improved forages in Sud-Kivu, DR Congo. Journal of Agriculture and Rural Development in the Tropics and Subtropics. https://doi.org/10.7910/DVN/HBO4EC
55. Reiber, C., Peters, M., Hoffmann, V., & Schultze-Kraft, R. (2012). Adoption and feeding of grass and legume hay in Honduras. Livestock Research for Rural Development, 24(11).
56. Rodríguez Portillo, S. A., Rodríguez Mejía, G. R., Marín Marín, Y. E., & Rodríguez Chávez, D. (2015). Feeding Cattle. Feeding cattle at critical times during the dry season in the Las Segovias area of Nicaragua. Catholic Relief Services. https://cgspace.cgiar.org/handle/10568/100774
57. Roothaert, R., & Kerridge, P. C. (2004). Adoption and scaling out: Strategies and experiences of the Forages for Smallholders Project. ITDG. https://cgspace.cgiar.org/handle/10568/75672
58. Sani, A. (2014). Socio-economic Factors Influencing Adoption of Dual-purpose Cowpea Production Technologies in Bichi Local Government Area of Kano State, Nigeria. Asian Journal of Agricultural Extension, Economics & Sociology, 3(4). https://doi.org/10.9734/ajaees/2014/7981
59. Selemani, I. S., Olav, E. L., Øystein, H., Ådnøy, T., Mtengeti, E., & Mushi, D. (2012). The role of indigenous knowledge and perceptions of pastoral communities on traditional grazing management in north-western Tanzania. African Journal of Agricultural Research, 7(40). https://doi.org/10.5897/AJAR12.1468
60. Shelton, H. M., Piggin, C. M., Acasio, R., Castillo, A., Mullen, B. F., Rika, I. K., Nulik, J., and Gutteridge, R. C. (1992). Case Studies of Locally-Successful Forage Tree Systems. In: Horne, P.,

Hacker, J. B., Kerridge, J. B., and Peter, C. (Eds.). Working with farmers: the key to adoption of forage technologies. Proceedings of an International Workshop held in Cagayan de Oro City, Mindanao, Philippines from 12-15 October 1992, pp. 120–131. ACIAR, Canberra, Australia.
61. Shiferaw, M., Asmare, B., Tegegne, F., & Molla, D. (2018). Farmers Perception and Utilization Status of Improved Forages Grown in the Natural Resource Areas of Northwestern Ethiopia. Biodiversitas 19(4), 1568–1578.
62. Sinja, J., Karugia, J. T., Mwangi, D. M., Baltenweck, I., & Romney, D. L. (2004, September 1). Fodder legumes technology and farmer to farmer extension: A case of Desmodium and Calliandra in central Kenya. Integrated Agricultural Research for Development-Achievements, Lessons Learnt and Best Practice. https://cgspace.cgiar.org/handle/10568/1768
63. Sinja, J., Nyangaga, J., Karugia, J. T., Waithaka, M. M., Mwangi, D. M., & Romney, D. L. (2004, November 8). Factors influencing farmer-to-farmer extension of forage legume technology. KARI Biennial Scientific Conference/Kenya Agricultural Research Forum, Nairobi, Kenya. https:/#/cgspace.cgiar.org/handle/10568/1847
64. Staal, S. J., Baltenweck, I., Waithaka, M. M., deWolf, T., & Njoroge, L. (2002). Location and Uptake: Integrated Household and GIS Analysis of Technology Adoption and Land Use, with Application to Smallholder Dairy Farms in Kenya. Agricultural Economics 27(3), 295–315.
65. Suman, M., Kumar, V., & Kumar, A. (2017). Study of farmers' perception constraints for lesser adoption of fodder production technologies and ways to improve the adoption in Bundelkhand region. Agricultural Science Digest - A Research Journal, 37(2). https://doi.org/10.18805/asd.v37i2.7997
66. Syomiti, M., Wanyoike, M., Wahome, R. G., & Kuria, J. K. N. (2011). The status of maize stover utilization as feed for livestock in Kiambu and Thika districts of Kenya: Constraints and opportuniti. https://pdfs.semanticscholar.org/5195/273bf8aef3ce4c5d4eb6edacef771c95f374.pdf?_ga=2.190629739.198011519.1578490222-2025453895.1578490222
67. Taylor-Powell, E., & Ingawa, S. A. (1986). Beneficiary Reactions to the Fodder Bank Trials. In: von Interactions between agronomy and economics in forage legume research. (1989). https://cgspace.cgiar.org/handle/10568/4569
68. Teufel, N., Johnson, N. and Singh, D. (2011). The adoption and impact of an improved drought-tolerant, dual-purpose groundnut variety in Southern India. Paper presented at the 7th ASAE International Conference 'Meeting the Challenges Facing Asian Agriculture and Agricultural Economics Toward a Sustainable Future', Hanoi, Vietnam, 13-15 October, 2011. Nairobi: ILRI. https://cgspace.cgiar.org/handle/10568/10587
69. Turinawe, A., Mugisha, J., & Kabirizibi, J. (2011). Socio-Economic Evaluation of Improved Forage Technologies in Smallholder Dairy Cattle Farming Systems in Uganda. Journal of Agricultural Science, 4(3). https://doi.org/10.5539/jas.v4n3p163
70. Nava-Tablada, M. E., Villa-Herrera, A., López-Ortiz, S., Vargas-López, S., Ortega-Jimenez, E., & López, F.-G. (2010). Use of Guacimo (Guazuma ulmifolia Lam.) as a forage source for extensive livestock production in a tropical area of Mexico. Tropical and Subtropical Agroecosystems, 10, 253–261. https://www.cabdirect.org/cabdirect/abstract/20093335904
71. Wambugu, C., Franzel, S., Tuwei, P., & Karanja, G. (2001). Scaling up the Use of Fodder Shrubs in Central Kenya. Development in Practice 11(4), 487–494.
72. Wanyama, J. M., Muyekho, F. N., Masinde, A. A. O., Cheruiyot, D. T., Odongo, J., Ojowi, M., & Okeyo, R. (2003). Assessing factors influencing adoption of pastures and fodders amongst smallholder subsistence farmers in selected districts of west Kenya. Tropical Grasslands, 37(4).

73. Zereu, G., and Lijalem, T. 2016. Status of Improved Forage Production, Utilization and Constraints for Adoption in Wolaita Zone, Southern Ethiopia. Livestock Research for Rural Development 28(5), 1–9.

Data extraction template

Type of publication; e.g. refereed journal

Notes; e.g. chapter 7 most relevant

Country of study; e.g. Kenya

Agroecological context; e.g. pastoral

Species; e.g. goat, sheep, cattle

Other; e.g. donkey

Feed option; e.g. Crop residue, Planted forage

Planted forage; y=1

Agroforestry; y=1

Crop residue; y=1

Other; e.g. natural grasslands

Cost model- public spending category; e.g. National R&D

Other; e.g. NGO

Qualitative, Quantitative, or Mixed?

Methodology; e.g. with/without

Other; e.g. survey among participants

Duration of 'experiment' in years; e.g. 4 years or not defined

Type of data used; e.g. survey

Quantitative : Does the study claim to statistically represent a particular population; e.g. district, region, country? y=1

What population (name and administrative unit)? e.g. Bure District, Ethiopia

Scale; e.g. household

Other; e.g. sites selected based on disease incidence (high medium low)

Sample Size; e.g. 120 families

Data Sources / Notes; e.g. Sample size does not seem realistic

Qualitative Data Type; e.g. Farm

Sample Size; e.g. 78 groups

Notes; e.g. random sample of cattle keepers

Outcomes – adoption, Level of measurement; e.g. community

Adoption; e.g. y=1

Other; e.g. Combined areas on 3 continents

Indicator used; e.g. % adoption

Change in indicator observed; e.g. 25% more compared to non-adopters

Outcomes – productivity, Level of measurement; e.g. animal

Other, Indicator used; e.g. dairy income; kids nutrition status

Indicator used; e.g. milk yield

Change in indicator observed; e.g. 25% more compared base feed option

Outcomes – livelihoods Level of measurement; e.g. family

Livelihood, y=1

Other; e.g. see 1st paragraph of survey design page 5

Indicator used; e.g. dairy income; kids nutrition status)

Change in indicator observed; e.g. 25% increase

Cost of Intervention; e.g. 6.58 USD per 500 trees

Does the paper claim to assess a policy, project, or other intervention? cut and paste if so

Are there any key findings related to sustainability; e.g. what factors contribute to adoption of good practices, …? Cut and paste if so

Are there findings related to drivers of adoption (positive effect on adoption)? List and /or cut and paste text

Are there findings related to constraints to adoption (negative effect on adoption)? List and /or cut and paste text

Protocol, including all final search strategies

Title: The impact of ruminant feed interventions on livelihoods among small scale and agro-pastoral livestock keepers in LMICs in Africa, Asia and Latin America: a scoping review protocol

Administrative Information

1. **Identification:** scoping review

2. **Registration:** https://osf.io/6ywht/

Submission on 5th June 2019 and amendment on 17th September 2019

3. **Authors**:

Corresponding author: Isabelle Baltenweck, International Livestock Research Institute, i.baltenweck@cgiar.org, PO Box 30709 Nairobi 00100 Kenya

Name	Organization	Country	Email	Role on the team
Isabelle Baltenweck	International Livestock Research Institute (ILRI)	Kenya	i.baltenweck@cgiar.or g	Lead author- livelihoods, adoption studies, gender
Steven J. Staal	International Livestock Research Institute (ILRI)	USA	s.staal@cgiar.org	Co author- livestock systems, economics- global
Elizaphan James O. Rao	International Livestock Research Institute (ILRI)	Kenya	J.Rao@cgiar.org	Co author- impact assessment, farm level economics- East Africa
Erin R. B. Eldermire	Cornell University	USA	erb29@cornell.edu	Research synthesis expert
Edda Tandi Lwoga	College of Business Education	Tanzania	tlwoga@gmail.com	Research synthesis expert
Nils Teufel	International Livestock Research Institute (ILRI)	Kenya	n.teufel@cgiar.org	Co author- livestock systems, impact assessment- South Asia, East Africa
Ricardo Labarta	CIAT	Colombi a	r.labarta@cgiar.org	Co author- feed and impact assessment Latin America

Alan Duncan	Edinburgh Uni and ILRI	UK	a.duncan@cgiar.org	Co author- feed expert and innovation systems
Debbie Cherney	Cornell University	USA	djc6@cornell.edu	Co-author- feed expert

Introduction

1. **Background:**

Livestock production supports the livelihoods of 1 billion poor people in Low and Middle Income Countries (LMICs) (Thorne and Conroy, 2017). Ruminant productivity is generally much lower in LMICs than in high income countries (e.g. Gerosa and Skoet, 2012). Livestock feed supply, in terms of quantity, quality and seasonality, is often described as the most important constraint to livestock productivity including for ruminants (Ayantunde et al, 2005, Thornton, 2010). Livestock feed improvement has therefore been the focus of considerable effort, by researchers, private and public sector and development agencies (e.g. NGOs) with a view to increasing ruminant productivity thus supporting smallholder development and improved farmer livelihoods (Devendra and Leng, 2011). Feed improvement interventions include introduction of improved grasses and legumes, methods of increasing intake and nutritive value of crop residues by physical or chemical treatment and methods for preserving fresh feed to fill the seasonal feed gap. Although 'improved' feed options have been researched and promoted widely, in many systems, by many entities (researchers etc), their uptake by farmers, their effects on ruminant productivity and ultimately, their impact on farmer livelihoods have not been systematically reviewed. This review therefore aims at scoping existing research publications to assess the extent of uptake of improved livestock feed options, the effect of this uptake on ruminant livestock productivity and the degree to which this improves smallholder farmer livelihoods. The overarching aim is to develop recommendations on the most promising feed options for improved productivity and ultimately farmer livelihoods, as well as identifying research gaps.

2. **Research question**:

What is the impact of ruminant feed interventions on livelihoods among small scale and agro-pastoral livestock keepers in LMICs in Africa, Asia and Latin America?

Methods

1. **Objectives:**

   a. **Setting**

The setting includes the LMICs in Africa, Asia and Latin America based on the World Bank classification scheme of countries (World Bank 2018). More precisely, we included low income, lower and upper middle income countries in East Asia & Pacific and South Asia, Latin America & Caribbean, Sub-Saharan

Africa, Middle East & North Africa. For East Asia & Pacific and South Asia, we selected all low income and lower middle income countries. For these two regions, we excluded all upper middle income countries except China.

b. Population

Small scale and agro- pastoral livestock keepers

c. Interventions

Intervention is defined as a plant- based livestock feed intervention. By plant-based interventions, we refer to actions involving plant-based raw materials such as forages and crop residues. Actions include introduction of novel planted forages, methods for crop residue improvement such as physical or chemical treatment, methods for sourcing feed such as lopping and cut-and-carry feeding; and methods for preserving fresh feed such as hay-making or ensiling to fill seasonal feed gaps. Industrial by-products and concentrates are excluded except where they are specifically used to improve crop residue quality e.g. addition of molasses to straw. Multiple interventions are defined as two or more single interventions that are applied to a single plant-based feed source. Institutional and market innovations, and information systems to promote dissemination and/or support adoption of these interventions are excluded as interventions, but impacts of respective interventions on institutional and market innovations could be included among potential outcomes. Treating plants with novelty industrial and/chemical treatments whose efficacy is not generally accepted by the scientific community are excluded.

d. Comparators

The review considers papers that describe the use of a feed technology (the intervention) and provides a comparison either between before and after intervention; or with and without intervention.

The intervention could have come from outside the system (e.g. researchers, private sector or NGOs promoting a new fodder variety or silage making) or have emerged endogenously (e.g. cattle keepers starting to feed crop residues), although most interventions have an external factor.

e. Outcome/s of interest

The outcomes of interest are categorised in three levels: 1. the adoption (or uptake) of the feed technology by farmers, either as a binary variable (yes/no) or a continuous variable for the extent of adoption (e.g. percentage of land in fodder); 2. the effect of the feed technology on animal productivity (e.g. forage use has increased milk production by 20%) and 3. the impact of the adoption of the feed technology on livelihood impact indicators, categorised as income (including earnings and proceeds), employment, food security and human nutrition.

One of the objectives of the review paper is to assess the extent to which research has focused on adoption studies, how many papers have reported effect of feed interventions on animal productivity, and thirdly how many papers have reported impact indicators of livelihoods.

f. **Study design/publication type**

The study design of the papers included in the literature review needs to be primary empirical research work, using quantitative, qualitative or mixed methods. Papers have to provide a comparison either between before and after intervention; or with and without intervention (defined as the feed intervention). Publication types include peer reviewed papers and grey literature, both qualitative and quantitative works

2. **Definitions:**

Low and middle income countries: We use the World Bank classification. We included low income, lower and upper middle income countries in East Asia & Pacific and South Asia, Latin America & Caribbean, Sub-Saharan Africa, Middle East & North Africa, and Middle East & North Africa. For East Asia & Pacific and South Asia, we selected all low income and lower middle income countries. For these two regions, we excluded all upper middle income countries except China.

Ruminants: Cattle and domesticated buffalos, sheep and goats, NOT camels, wild buffalos, yacks, alpacas

Small scale livestock keepers (focusing on ruminants): There is no standard definition of small scale as it depends on the species and location- but usually 1 to 5 heads of cattle (but higher for Latin America) and below 10 head for sheep and goats.

Agro-pastoral livestock keepers (focusing on ruminants): Households keeping small and/or large ruminants, deriving a livelihood from both crop and livestock (so no pure pastoralists) and practicing grazing and part time transhumance.

Ruminant feed strategies or interventions: Plant-based feed interventions (forage, crop residues, their processing including conservation) but excluding industrial by-products and concentrates.

Outcomes:

a) **uptake/adoption**: Uptake/adoption of feed strategies or interventions. The use of feed strategies or interventions, either as binary (yes/no) variable, the extent of adoption, as a one-off or continuous decision; diffusion beyond target farmers
b) **Animal productivity**: Effect of feed strategies or interventions on animal productivity. Depending on species, these may contain: milk yield, weight gain, reproductive performance, herd offtake.
c) **Livelihoods**: Impact of the feed strategies or interventions on livelihoods. Indicators could include impact on income from livestock (including earnings and proceeds), labour saving, employment in the livestock and related value chains, food security and human nutrition

3. **Eligibility criteria**:

For an article to be included in this study, it must meet all of the following inclusion criteria:

1. Study focus includes population of small-scale and agro pastoral keepers of large and small ruminants
2. Primary empirical research
3. Explicit population focus is small-scale and agro pastoral ruminant livestock keepers
4. Study describes the adoption of 'improved feed options'; in addition to adoption, the effect on productivity and/or their impact on livelihoods
5. Study area or focus includes target populations in low and middle income countries
6. Study is in English, French, Spanish or German

For an article to be excluded from this scoping review, it must meet one of the following exclusion criteria:

1. Study that is a review or a case study
2. Study does not include small-scale or agro pastoral as the target population
3. Study does not take place in our targeted countries (small and middle income countries) in Latin America, Africa or Asia
4. Study does not consider improved feed options (introduced by external entity or farmer own experimentation). Note: "improved" can mean options that have not previously been used in the study site
5. Study only looks at industrial by-products and/or concentrates
6. Study is in a language other than English, French, Spanish or German
7. Study that only consider fish, pigs, poultry, camels, wild buffalos, yaks, alpacas, guinea pigs (or cavies), bees, equines, rabbits, all wildlife animals

4. **Information sources:**

Two research synthesis experts will conduct searches of the following electronic databases:

- CAB Abstracts (access via Web of Science)
- Web of Science Core Collection (access via Web of Science)
- Scopus (access via Elsevier)

A search of the following grey literature sources will also be conducted:

- Dissertations and Theses Global (access via ProQuest)
- Africa Theses and Dissertations
- AgEcon Search
- AGRIS (FAO-consolidated search)

- Campbell Collaboration
- Cochrane Collaboration
- Collaboration for Environmental Evidence (CEE)
- Commonwealth Scientific and Industrial Research Organisation (CSIRO)
- EMBRAPA
- French Agricultural Research Centre for International Development (CIRAD)
- Gardian (searches 15 CGIAR websites)
- International Fund for Agricultural Development (IFAD)
- International Institute for Environment and Development
- JPAL/ATAI impact evaluations (IPA)
- Overseas Development Institute (ODI)
- UK Department for International Development (DFID)
- UNEP
- WFP
- WHO
- World Bank
- Cgspace

In addition, we will also screen through the list of references of included studies for additional relevant studies that we may have missed during the search process.

5. **Search strategy:**

A comprehensive search strategy has been developed (TL and ERBE) to identify all available research pertaining to the livelihoods of small scale and agro-pastoral livestock keepers in low- and middle-income countries in Africa, Asia and Latin America, in relation to the improvement of ruminant feed interventions. Search terms will include variations of the key concepts in the research question: improvement or conservation of crops produced for ruminants, small-scale producers and pastoralists, low- and middle-income countries in Africa, Asia and Latin America, and innovation or livelihood indicators. See Appendix A for a presentation of the search strategy in its entirety such that it may be reproduced in CAB Abstracts (accessed via the Web of Science platform).

6. **Study records**

    a. **Data management:**

Searches will be performed across all sources listed in section 5 of this protocol, and the search results will be de-duplicated to remove redundant citations identified from multiple sources. Titles, abstracts, and keywords of all citations will be exported as .RIS files. We will use machine learning processes to accelerate the screening process. This includes adding machine-derived metadata to the individual citations, such as identifying populations, geographies, interventions, and outcomes of interest. This will allow for accelerated identification of potential articles for exclusion at the title/abstract screening stage.

    b. **Selection process:**

Article screening will take place in three phases:

1. With the machine learning as described in section 6a, an initial title and abstract review will be performed blindly in Excel by author pairs. At this first stage, each author will only note whether a citation should be excluded. Author pairs will compare results, and only citations for which both authors agreed that a citation be excluded will be excluded. All citations for which a conflict exists will be transferred to the next stage.
2. Remaining citations (including the conflicts from screening step 1, above) will be uploaded to Covidence for title and abstract screening. Citations will be blindly screened for relevance by two authors against the inclusion and exclusion criteria listed in section 4 of this protocol. Conflicts will go to a third reviewer to break ties.
3. Full-text screening of all articles deemed relevant in the title and abstract screening phase will occur. Citations will be screened for relevance against the inclusion and exclusion criteria listed in section 4 of this protocol. Reasons for exclusion will be documented within Covidence.

In both phases of Covidence screening, all citations will be reviewed for relevance by two independent reviewers (IB, SS, ER, MB, NT, RL, AD, DC, EE). Each citation that meets all of the inclusion criteria at the title, abstract, and full-text screening phases will be included. Each citation that meets one of the exclusion criteria at the title and abstract and full-text screening phases will be excluded. All conflicts will be resolved in Covidence by a third, independent reviewer.

c. Data collection process:

We will develop a data extraction template to document all themes of interest listed in section 9 of this protocol (Data Synthesis) for each included study. Data extraction form will have information such as:

- Author(s), year of publication, study location
- Intervention type, and comparator (if any); duration of the intervention
- Study population
- Aims of the study
- Methodology
- Outcome measures
- Important results

This data plus any additional standardized data will be entered into a database and will form the basis of the analysis. The data extraction template will be tested by the review team before use, and data will be extracted by independent reviewers for each study.

7. Critical appraisal of individual sources of evidence:

The proposed evidence synthesis will be informed by methodological guidelines for scoping reviews (Arksey and O'Malley, 2006; Levac et al 2010). For this scoping review, and due to the expected heterogeneity in study design of the included studies, we may assess individual sources of evidence for methodological quality or risk of bias, however we will not perform a formal critical appraisal of all included studies. Instead we will perform selective critical appraisal of groups of similar study types identified within included studies, using a tool final adapted from the Critical Appraisal Skills Programme

(CASP) protocol (see https://casp-uk.net/casp-tools-checklists/) Quality of data assessment may include appraisal of study replicability, multi-year, multi-location, size of sample population. The final manuscript will include an explanation of how the appraisal aligns with the review methods.

8. Data synthesis/charting:

Since the scoping review presents an overview of all the material that has been reviewed, there is a need to consider how best to present the potentially large body of literature in a manner that makes sense to readers. We will undertake this through a 3-step process as follows:

- Undertake basic numerical analysis of the extent, nature and distribution of studies included in the review. This will be achieved via tables and charts that map distribution of studies geographically, by range of interventions, types of outcomes (adoption, animal productivity or livelihood indicators), research methods adopted and measure of effectiveness based on the results from the critical appraisal. This helps in highlighting dominant areas of research in terms of intervention types, research methods and geographical location; and also, where the research gaps are.
- Identify thematic area for organizing the literature. For this review, the key thematic area, which is also the primary unit of analysis could be intervention type. Primary studies will therefore be organized into intervention categories.
- Provide a consistent approach to reporting our findings and develop a template that we apply to each intervention group/categories. The template will include:
    - A table that summarizes basic characteristics of all studies in every intervention group/category
    - Commentary/narrative for each intervention group under the following sub-headings; Interventions, sample size, participants, research methods, outcomes, evidence of effectiveness, economic aspects, study gaps
- To ensure that evidence is captured accurately, data from each included study will be extracted by one author and reviewed by a second author.

References

Arksey, H. and O'Malley, L. 2005. Scoping studies: towards a methodological framework. International Journal of Social Research Methodology. 8(1), pp. 19-32.

Ayantunde, A.A., Fernández-Rivera, S. and McCrabb, G.J., 2005. Coping with feed scarcity in smallholder livestock systems in developing countries. ILRI.

Campbell Library. (n.d.). Retrieved March 6, 2019, from https://campbellcollaboration.org/library/campbell-systematic-review-templates.html

Devendra, C. and Leng, R.A., 2011. Feed resources for animals in Asia: issues, strategies for use, intensification and integration for increased productivity. Asian-Australasian Journal of Animal Sciences, 24(3), pp.303-321.

Gerosa, S. and Skoet, J., 2012. Milk availability: trends in production and demand and medium-term outlook (No. 289000). Food and Agriculture Organization of the United Nations, Agricultural Development Economics Division (ESA).

Levac, D., Colquhoun, H., and O'Brien, K. K. 2010. Scoping studies: advancing the methodology. Implementation Science, 5(69), 1-9. https://doi.org/10.1186/1748-5908-5-69

PRISMA-P-checklist.pdf. (n.d.). Retrieved from http://www.prisma-statement.org/documents/PRISMA-P-checklist.pdf

Thorne, P., & Conroy, C. (2017). Research on Livestock, Livelihoods, and Innovation. In *Agricultural Systems* (pp. 303-330). Academic Press.

Thornton, P. K. (2010). Livestock production: recent trends, future prospects. *Philosophical Transactions of the Royal Society B: Biological Sciences*, *365*(1554), 2853-2867.

Tricco, A. C., Lillie, E., Zarin, W., O'Brien, K. K., Colquhoun, H., Levac, D., … Straus, S. E. (2018). PRISMA Extension for Scoping Reviews (PRISMA-ScR): Checklist and Explanation. *Annals of Internal Medicine*, *169*(7), 467. https://doi.org/10.7326/M18-0850

World Bank (2018). World Bank Country and Lending Groups. https://datahelpdesk.worldbank.org/knowledgebase/articles/906519

Appendix A

Row #	Search string
1	TS=("American Samoa" OR "China" OR "Fiji" OR "Micronesia" OR "Indonesia" OR "Cambodia" OR "Kiribati" OR "Lao" OR "Marshall Islands" OR "Myanmar" OR "Mongolia" OR "Malaysia" OR "Nauru" OR "Philippines" OR "Papua New Guinea" OR "Korea" OR "Solomon Islands" OR "Thailand" OR "Timor-Leste" OR "Tonga" OR "Tuvalu" OR "Vietnam" OR "Vanuatu" OR "Samoa")
2	TS=("Belize" OR "Bolivia" OR "Brazil" OR "Colombia" OR "Costa Rica" OR "Cuba" OR "Dominica" OR "Dominican Republic" OR "Ecuador" OR "Grenada" OR "Guatemala" OR "Guyana" OR "Honduras" OR "Haiti" OR "Jamaica" OR "St. Lucia" OR "Mexico" OR "Nicaragua" OR "Peru" OR "Paraguay" OR "El Salvador" OR "Suriname" OR "St. Vincent and the Grenadines" OR "Venezuela")
3	TS=("Djibouti" OR "Algeria" OR "Egypt" OR "Iran" OR "Iraq" OR "Jordan" OR "Lebanon" OR "Libya" OR "Morocco" OR "West Bank" OR "Gaza" OR "Syrian Arab Republic" OR "Tunisia" OR "Yemen")
4	TS=("Angola" OR "Benin" OR "Botswana" OR "Burkina Faso" OR "Burundi" OR "Cameroon" OR "Cape Verde" OR "Cabo Verde" OR "Central African Republic" OR "Chad" OR "Tchad" OR "Comoros" OR "Congo" OR "Cote d'Ivoire" OR "Ivory Coast" OR "Djibouti" OR "Equatorial Guinea" OR "Eritrea" OR "Ethiopia" OR "Gabon" OR "Gambia" OR "Ghana" OR "Guinea" OR "Kenya" OR "Lesotho" OR "Liberia" OR "Madagascar" OR "Malawi" OR "Mali" OR "Mauritania" OR "Mauritius" OR "Mozambique" OR "Namibia" OR "Niger" OR "Nigeria" OR "Réunion" OR "Rwanda" OR "Sao Tome and Principe" OR "Senegal" OR "Seychelles" OR "Sierra Leone" OR "Somalia" OR "South Africa" OR "Sudan" OR "Swaziland" OR "Swasiland" OR "Eswatini" OR

	"Tanzania" OR "Togo" OR "Uganda" OR "Western Sahara" OR "Zaire" OR "Zambia" OR "Zimbabwe" OR "Rhodesia")
5	TS=("Afghanistan" OR "Bangladesh" OR "Bhutan" OR "India" OR "Maldives" OR "Nepal" OR "Pakistan" OR "Sri Lanka")
6	#1 OR #2 OR #3 OR #4 OR #5
7	TS=("developing" OR "less-developed" OR "under-developed" OR "underdeveloped" OR "middle income" OR "low income" OR "underserved" OR "under served" OR "deprived" OR "poor")
8	TS=("countr*" OR "nation*" OR "population*" OR "world")
9	#7 AND #8
10	TS=("developing" OR "less-developed" OR "under developed" OR "underdeveloped" OR "low gdp" OR "low gnp" OR "low gross domestic" OR "low gross national")
11	TS=("economy" or "economies")
12	#10 AND #11
13	TS=("lmic" OR "lmics" OR "third world" OR "lami country" OR "lami countries" OR "transitional country" OR "transitional countries")
14	#9 OR #12 OR #13
15	#6 OR #14
16	TS=("smallhold*" OR "small hold*" OR "small farm*" OR "microfarm*" OR "micro-farm*" OR "pastoral" OR "family-run farm*" OR "family-owned farm*" OR "family-managed farm*" OR "agropastoral" OR "agro-pastoral" OR "ejido")
17	TS=(("small-scale" OR "smallscale" OR "low-income" OR "subsistence" OR "semi-subsistence" OR "resource-poor" OR "resource-limited" OR "small-size*" OR "low-income" OR "peasant" OR "district*" OR "village" OR "local" OR "household*") NEAR/3 ("farm*" OR "mixed-farm*" OR "agriculture" OR "producer*" OR "grower*" OR "agronomy" OR "husbandry"))
18	DE=("smallholders" OR "small farms" OR "pastoralism" OR "agropastoral systems" OR "silvopastoral systems" OR "livestock farming" OR "community involvement" OR "mixed farming")
19	#16 OR #17 OR #18
20	TS=("multipurpose tree*" OR "multipurpose crop*" OR "fallow*" OR "total mixed ration*" OR "forag*" OR "fodder" OR "leguminous shrub*" OR "cut-and-carry" OR "tree lopping" OR "fodder")
21	TS=("hay" OR “silage" OR “ensil*" OR “bale" OR “baling" OR "baler" OR “acetylation" OR “electrolysis" OR “esterification" OR “*hydration" OR “hydrogenation" OR “iodination" OR “neutralization" OR “tanning" OR “hay-making")

22	TS=("crop residue*" OR "roughage" OR "stover" OR “straw")
23	TS=((“osmo*" OR “chemical" OR “acid*" OR “alkali*" OR “ammonia" OR “ion exchange" OR “sodium hydroxide") Near/2 (“*treat*"))
24	TS= (“chopping" OR “chemical treatment" OR “urea" OR “pulveriz*" OR “supplement*" OR “feed* block*")
25	#23 OR #24
26	#22 AND #25
27	DE=("crop residues" OR "stover" OR "improved fallow" OR "foraging" OR "forage" OR "fodder crops" OR "silage" OR “chemical treatment")
28	#20 OR #21 OR #26 OR #27
29	TS=("adopt*" OR "enact*" OR "adapt*" OR "apply" OR "applies" OR "application*" OR "innovat*" OR "uptake" OR "use" OR "usage" OR "using" OR "utiliz*")
30	TS=("income" OR "employ*" OR "compensat*" OR "earn*" OR "livelihood*" OR "pay" OR "payment*" OR "proceed" OR "proceeds" OR "profit*" OR "salar*" OR "harvest*" OR "productiv*" OR "income*" OR "revenue*" OR "wage*" OR "subsistence" OR "enterprise" OR "food secur*" OR "human nutrition" OR "on-farm" OR "off-farm" OR "labor" OR "labour" OR "value add*" OR "improv*" OR "soil fertility" OR "soil exhaustion" OR "socioeconomic*" OR “socio-economic*")
31	DE=("innovation adoption" OR "innovations" OR "income" OR "productivity" OR "value added" OR "profitability" OR "livelihoods" OR "improvement" OR "soil fertility" OR "soil exhaustion" OR "socioeconomics" OR "food security" OR "employment")
32	#29 OR #30 OR #31
33	#15 AND #19 AND #28 AND #32

All search strategies

The impact of ruminant feed interventions on livelihoods among small scale and agro-pastoral livestock keepers in LMICs in Africa, Asia and Latin America: a scoping review

Search strategy for CAB Abstracts and Global Health (Web of Science)

###Searches performed May 24, 2019 (updated October 9, 2019)

####Number of records retrieved n=10,262

1. TS=("American Samoa" OR "China" OR "Fiji" OR "Micronesia" OR "Indonesia" OR "Cambodia" OR "Kiribati" OR "Lao" OR "Marshall Islands" OR "Myanmar" OR "Mongolia" OR "Malaysia" OR "Nauru" OR "Philippines" OR "Papua New Guinea" OR "Korea" OR "Solomon Islands" OR "Thailand" OR "Timor-Leste" OR "Tonga" OR "Tuvalu" OR "Vietnam" OR "Vanuatu" OR "Samoa" OR “Belize" OR "Bolivia" OR "Brazil" OR "Colombia" OR "Costa Rica" OR "Cuba" OR "Dominica" OR "Dominican Republic" OR "Ecuador" OR "Grenada" OR "Guatemala" OR "Guyana" OR "Honduras" OR "Haiti" OR "Jamaica" OR "St. Lucia" OR "Mexico" OR "Nicaragua" OR "Peru" OR "Paraguay" OR "El Salvador" OR "Suriname" OR "St. Vincent and the Grenadines" OR "Venezuela" OR “Djibouti" OR "Algeria" OR "Egypt" OR "Iran" OR "Iraq" OR "Jordan" OR "Lebanon" OR "Libya" OR "Morocco" OR "West Bank" OR "Gaza" OR "Syrian Arab Republic" OR "Tunisia" OR "Yemen" OR “Angola" OR "Benin" OR "Botswana" OR "Burkina Faso" OR "Burundi" OR "Cameroon" OR "Cape Verde" OR "Cabo Verde" OR "Central African Republic" OR "Chad" OR "Tchad" OR "Comoros" OR "Congo" OR "Cote d’Ivoire" OR "Ivory Coast" OR "Djibouti" OR "Equatorial Guinea" OR "Eritrea" OR "Ethiopia" OR "Gabon" OR "Gambia" OR "Ghana" OR "Guinea" OR "Kenya" OR "Lesotho" OR "Liberia" OR "Madagascar" OR "Malawi" OR "Mali" OR "Mauritania" OR "Mauritius" OR "Mozambique" OR "Namibia" OR "Niger" OR "Nigeria" OR "Réunion" OR "Rwanda" OR "Sao Tome and Principe" OR "Senegal" OR "Seychelles" OR "Sierra Leone" OR "Somalia" OR "South Africa" OR "Sudan" OR "Swaziland" OR "Swasiland" OR "Eswatini" OR "Tanzania" OR "Togo" OR "Uganda" OR "Western Sahara" OR "Zaire" OR "Zambia" OR "Zimbabwe" OR "Rhodesia" OR ”Afghanistan" OR "Bangladesh" OR "Bhutan" OR "India" OR "Maldives" OR "Nepal" OR "Pakistan" OR "Sri Lanka")
2. TS=("developing" OR "less-developed" OR "under-developed" OR "underdeveloped" OR "middle income" OR "low income" OR "underserved" OR "under served" OR "deprived" OR "poor")
3. TS=("countr*" OR "nation*" OR "population*" OR "world")
4. #2 AND #3
5. TS=("developing" OR "less-developed" OR "under developed" OR "underdeveloped" OR "low gdp" OR "low gnp" OR "low gross domestic" OR "low gross national")
6. TS=("economy" or "economies")
7. #5 AND #6
8. TS=("lmic" OR "lmics" OR "third world" OR "lami country" OR "lami countries" OR "transitional country" OR "transitional countries")
9. #4 OR #7 OR #8
10. #1 OR #9
11. TS=("smallhold*" OR "small hold*" OR "small farm*" OR "microfarm*" OR "micro-farm*" OR "pastoral" OR "family-run farm*" OR "family-owned farm*" OR "family-managed farm*" OR "agropastoral" OR "agro-pastoral" OR "ejido")

12. TS=(("small-scale" OR "smallscale" OR "low-income" OR "subsistence" OR "semi-subsistence" OR "resource-poor" OR "resource-limited" OR "small-size*" OR "low-income" OR "peasant" OR "district*" OR "village" OR "local" OR "household*") NEAR/3 ("farm*" OR "mixed-farm*" OR "agriculture" OR "producer*" OR "grower*" OR "agronomy" OR "husbandry"))
13. DE=("smallholders" OR "small farms" OR "pastoralism" OR "agropastoral systems" OR "silvopastoral systems" OR "livestock farming" OR "community involvement" OR "mixed farming")
14. #11 OR #12 OR #13
15. TS=("multipurpose tree*" OR "multipurpose crop*" OR "fallow*" OR "total mixed ration*" OR "forag*" OR "fodder" OR "leguminous shrub*" OR "cut-and-carry" OR "tree lopping" OR "fodder" OR ”hay" OR “silage" OR “ensil*" OR “bale" OR “baling" OR "baler" OR “acetylation" OR “electrolysis" OR “esterification" OR “*hydration" OR “hydrogenation" OR “iodination" OR “neutralization" OR “tanning" OR “hay-making")
16. TS=("crop residue*" OR "roughage" OR "stover" OR “straw")
17. TS=((“osmo*" OR “chemical" OR “acid*" OR “alkali*" OR “ammonia" OR “ion exchange" OR “sodium hydroxide") Near/2 (“*treat*"))
18. TS= (“chopping" OR “chemical treatment" OR “urea" OR “pulveriz*" OR “supplement*" OR “feed* block*")
19. #17 OR #18
20. #16 AND #19
21. DE=("crop residues" OR "stover" OR "improved fallow" OR "foraging" OR "forage" OR "fodder crops" OR "silage" OR “chemical treatment")
22. #15 OR #20 OR #21
23. TS=("adopt*" OR "enact*" OR "adapt*" OR "apply" OR "applies" OR "application*" OR "innovat*" OR "uptake" OR "use" OR "usage" OR "using" OR "utiliz*” OR "income" OR "employ*" OR "compensat*" OR "earn*" OR "livelihood*" OR "pay" OR "payment*" OR "proceed" OR "proceeds" OR "profit*" OR "salar*" OR "harvest*" OR "productiv*" OR "income*" OR "revenue*" OR "wage*" OR "subsistence" OR "enterprise" OR "food secur*" OR "human nutrition" OR "on-farm" OR "off-farm" OR "labor" OR "labour" OR "value add*" OR "improv*" OR "soil fertility" OR "soil exhaustion" OR "socioeconomic*" OR “socio-economic*")
24. DE=("innovation adoption" OR "innovations" OR "income" OR "productivity" OR "value added" OR "profitability" OR "livelihoods" OR "improvement" OR "soil fertility" OR "soil exhaustion" OR "socioeconomics" OR "food security" OR "employment")
25. #23 OR #24
26. #10 AND #14 AND #22 AND #25

The impact of ruminant feed interventions on livelihoods among small scale and agro-pastoral livestock keepers in LMICs in Africa, Asia and Latin America: a scoping review

##Search strategy for Web of Science Core Collection (Web of Science)

###Searches performed May 24, 2019 (updated October 9, 2019)

####Number of records retrieved n=1,421

1. TS=("American Samoa" OR "China" OR "Fiji" OR "Micronesia" OR "Indonesia" OR "Cambodia" OR "Kiribati" OR "Lao" OR "Marshall Islands" OR "Myanmar" OR "Mongolia" OR "Malaysia" OR "Nauru" OR "Philippines" OR "Papua New Guinea" OR "Korea" OR "Solomon Islands" OR "Thailand" OR "Timor-Leste" OR "Tonga" OR "Tuvalu" OR "Vietnam" OR "Vanuatu" OR "Samoa" OR ″Belize" OR "Bolivia" OR "Brazil" OR "Colombia" OR "Costa Rica" OR "Cuba" OR "Dominica" OR "Dominican Republic" OR "Ecuador" OR "Grenada" OR "Guatemala" OR "Guyana" OR "Honduras" OR "Haiti" OR "Jamaica" OR "St. Lucia" OR "Mexico" OR "Nicaragua" OR "Peru" OR "Paraguay" OR "El Salvador" OR "Suriname" OR "St. Vincent and the Grenadines" OR "Venezuela" OR ″Djibouti" OR "Algeria" OR "Egypt" OR "Iran" OR "Iraq" OR "Jordan" OR "Lebanon" OR "Libya" OR "Morocco" OR "West Bank" OR "Gaza" OR "Syrian Arab Republic" OR "Tunisia" OR "Yemen" OR ″Angola" OR "Benin" OR "Botswana" OR "Burkina Faso" OR "Burundi" OR "Cameroon" OR "Cape Verde" OR "Cabo Verde" OR "Central African Republic" OR "Chad" OR "Tchad" OR "Comoros" OR "Congo" OR "Cote d’Ivoire" OR "Ivory Coast" OR "Djibouti" OR "Equatorial Guinea" OR "Eritrea" OR "Ethiopia" OR "Gabon" OR "Gambia" OR "Ghana" OR "Guinea" OR "Kenya" OR "Lesotho" OR "Liberia" OR "Madagascar" OR "Malawi" OR "Mali" OR "Mauritania" OR "Mauritius" OR "Mozambique" OR "Namibia" OR "Niger" OR "Nigeria" OR "Réunion" OR "Rwanda" OR "Sao Tome and Principe" OR "Senegal" OR "Seychelles" OR "Sierra Leone" OR "Somalia" OR "South Africa" OR "Sudan" OR "Swaziland" OR "Swasiland" OR "Eswatini" OR "Tanzania" OR "Togo" OR "Uganda" OR "Western Sahara" OR "Zaire" OR "Zambia" OR "Zimbabwe" OR "Rhodesia" OR ″Afghanistan" OR "Bangladesh" OR "Bhutan" OR "India" OR "Maldives" OR "Nepal" OR "Pakistan" OR "Sri Lanka")
2. TS=("developing" OR "less-developed" OR "under-developed" OR "underdeveloped" OR "middle income" OR "low income" OR "underserved" OR "under served" OR "deprived" OR "poor")
3. TS=("countr*" OR "nation*" OR "population*" OR "world")
4. #2 AND #3
5. TS=("developing" OR "less-developed" OR "under developed" OR "underdeveloped" OR "low gdp" OR "low gnp" OR "low gross domestic" OR "low gross national")
6. TS=("economy" or "economies")
7. #5 AND #6
8. TS=("lmic" OR "lmics" OR "third world" OR "lami country" OR "lami countries" OR "transitional country" OR "transitional countries")
9. #4 OR #7 OR #8
10. #1 OR #9
11. TS=("smallhold*" OR "small hold*" OR "small farm*" OR "microfarm*" OR "micro-farm*" OR "pastoral" OR "family-run farm*" OR "family-owned farm*" OR "family-managed farm*" OR "agropastoral" OR "agro-pastoral" OR "ejido")
12. TS=(("small-scale" OR "smallscale" OR "low-income" OR "subsistence" OR "semi-subsistence" OR "resource-poor" OR "resource-limited" OR "small-size*" OR "low-income" OR "peasant" OR "district*" OR "village" OR "local" OR "household*") NEAR/3 ("farm*" OR "mixed-farm*" OR "agriculture" OR "producer*" OR "grower*" OR "agronomy" OR "husbandry"))

13. #11 OR #12
14. TS=("multipurpose tree*" OR "multipurpose crop*" OR "fallow*" OR "total mixed ration*" OR "forag*" OR "fodder" OR "leguminous shrub*" OR "cut-and-carry" OR "tree lopping" OR "fodder" OR "hay" OR "silage" OR "ensil*" OR "bale" OR "baling" OR "baler" OR "acetylation" OR "electrolysis" OR "esterification" OR "*hydration" OR "hydrogenation" OR "iodination" OR "neutralization" OR "tanning" OR "hay-making")
15. TS=("crop residue*" OR "roughage" OR "stover" OR "straw")
16. TS=(("osmo*" OR "chemical" OR "acid*" OR "alkali*" OR "ammonia" OR "ion exchange" OR "sodium hydroxide") Near/2 ("*treat*"))
17. TS= ("chopping" OR "chemical treatment" OR "urea" OR "pulveriz*" OR "supplement*" OR "feed* block*")
18. #16 OR #17
19. #15 AND #18
20. #14 OR #19
21. TS=("adopt*" OR "enact*" OR "adapt*" OR "apply" OR "applies" OR "application*" OR "innovat*" OR "uptake" OR "use" OR "usage" OR "using" OR "utiliz*" OR ”income" OR "employ*" OR "compensat*" OR "earn*" OR "livelihood*" OR "pay" OR "payment*" OR "proceed" OR "proceeds" OR "profit*" OR "salar*" OR "harvest*" OR "productiv*" OR "income*" OR "revenue*" OR "wage*" OR "subsistence" OR "enterprise" OR "food secur*" OR "human nutrition" OR "on-farm" OR "off-farm" OR "labor" OR "labour" OR "value add*" OR "improv*" OR "soil fertility" OR "soil exhaustion" OR "socioeconomic*" OR "socio-economic*")
22. #10 AND #13 AND #20 AND #21

The impact of ruminant feed interventions on livelihoods among small scale and agro-pastoral livestock keepers in LMICs in Africa, Asia and Latin America: a scoping review

Search strategy for Scopus (Elsevier)

###Searches performed May 24, 2019 (updated October 9, 2019)

####Number of records retrieved n=1,171

1. TITLE-ABS-KEY ("American Samoa" OR "China" OR "Fiji" OR "Micronesia" OR "Indonesia" OR "Cambodia" OR "Kiribati" OR "Lao" OR "Marshall Islands" OR "Myanmar" OR "Mongolia" OR "Malaysia" OR "Nauru" OR "Philippines" OR "Papua New Guinea" OR "Korea" OR "Solomon Islands" OR "Thailand" OR "Timor-Leste" OR "Tonga" OR "Tuvalu" OR "Vietnam" OR "Vanuatu" OR "Samoa")
2. TITLE-ABS-KEY ("Belize" OR "Bolivia" OR "Brazil" OR "Colombia" OR "Costa Rica" OR "Cuba" OR "Dominica" OR "Dominican Republic" OR "Ecuador" OR "Grenada" OR "Guatemala" OR "Guyana" OR "Honduras" OR "Haiti" OR "Jamaica" OR "St. Lucia" OR "Mexico" OR "Nicaragua" OR "Peru" OR "Paraguay" OR "El Salvador" OR "Suriname" OR "St. Vincent and the Grenadines" OR "Venezuela")
3. TITLE-ABS-KEY ("Djibouti" OR "Algeria" OR "Egypt" OR "Iran" OR "Iraq" OR "Jordan" OR "Lebanon" OR "Libya" OR "Morocco" OR "West Bank" OR "Gaza" OR "Syrian Arab Republic" OR "Tunisia" OR "Yemen")
4. TITLE-ABS-KEY ("Angola" OR "Benin" OR "Botswana" OR "Burkina Faso" OR "Burundi" OR "Cameroon" OR "Cape Verde" OR "Cabo Verde" OR "Central African Republic" OR "Chad" OR "Tchad" OR "Comoros" OR "Congo" OR "Cote d'Ivoire" OR "Ivory Coast" OR "Djibouti" OR "Equatorial Guinea" OR "Eritrea" OR "Ethiopia" OR "Gabon" OR "Gambia" OR "Ghana" OR "Guinea" OR "Kenya" OR "Lesotho" OR "Liberia" OR "Madagascar" OR "Malawi" OR "Mali" OR "Mauritania" OR "Mauritius" OR "Mozambique" OR "Namibia" OR "Niger" OR "Nigeria" OR "Réunion" OR "Rwanda" OR "Sao Tome and Principe" OR "Senegal" OR "Seychelles" OR "Sierra Leone" OR "Somalia" OR "South Africa" OR "Sudan" OR "Swaziland" OR "Swasiland" OR "Eswatini" OR "Tanzania" OR "Togo" OR "Uganda" OR "Western Sahara" OR "Zaire" OR "Zambia" OR "Zimbabwe" OR "Rhodesia")
5. TITLE-ABS-KEY ("Afghanistan" OR "Bangladesh" OR "Bhutan" OR "India" OR "Maldives" OR "Nepal" OR "Pakistan" OR "Sri Lanka")
6. #1 OR #2 OR #3 OR #4 OR #5
7. TITLE-ABS-KEY ("developing" OR "less-developed" OR "under-developed" OR "underdeveloped" OR "middle income" OR "low income" OR "underserved" OR "under served" OR "deprived" OR "poor")
8. TITLE-ABS-KEY ("countr*" OR "nation*" OR "population*" OR "world")
9. #7 AND #8
10. TITLE-ABS-KEY ("developing" OR "less-developed" OR "under developed" OR "underdeveloped" OR "low gdp" OR "low gnp" OR "low gross domestic" OR "low gross national")
11. TITLE-ABS-KEY ("economy" or "economies")
12. #10 AND #11
13. TITLE-ABS-KEY ("lmic" OR "lmics" OR "third world" OR "lami country" OR "lami countries" OR "transitional country" OR "transitional countries")
14. #9 OR #12 OR #13

15. #6 OR #14
16. TITLE-ABS-KEY ("smallhold*" OR "small hold*" OR "small farm*" OR "microfarm*" OR "micro-farm*" OR "pastoral" OR "family-run farm*" OR "family-owned farm*" OR "family-managed farm*" OR "agropastoral" OR "agro-pastoral" OR "ejido")
17. TITLE-ABS-KEY (("small-scale" OR "smallscale" OR "low-income" OR "subsistence" OR "semi-subsistence" OR "resource-poor" OR "resource-limited" OR "small-size*" OR "low-income" OR "peasant" OR "district*" OR "village" OR "local" OR "household*") NEAR/3 ("farm*" OR "mixed-farm*" OR "agriculture" OR "producer*" OR "grower*" OR "agronomy" OR "husbandry"))
18. #16 OR #17
19. TITLE-ABS-KEY ("multipurpose tree*" OR "multipurpose crop*" OR "fallow*" OR "total mixed ration*" OR "forag*" OR "fodder" OR "leguminous shrub*" OR "cut-and-carry" OR "tree lopping" OR "fodder")
20. TITLE-ABS-KEY ("hay" OR “silage" OR “ensil*" OR “bale" OR “baling" OR "baler" OR “acetylation" OR “electrolysis" OR “esterification" OR “*hydration" OR “hydrogenation" OR “iodination" OR “neutralization" OR “tanning" OR “hay-making")
21. TITLE-ABS-KEY ("crop residue*" OR "roughage" OR "stover" OR “straw")
22. TITLE-ABS-KEY ((“osmo*" OR “chemical" OR “acid*" OR “alkali*" OR “ammonia" OR “ion exchange" OR “sodium hydroxide") Near/2 (“*treat*”))
23. TITLE-ABS-KEY (“chopping" OR “chemical treatment" OR “urea" OR “pulveriz*" OR “supplement*" OR “feed* block*")
24. #22 OR #23
25. #21 AND #24
26. #19 OR #20 OR #25
27. TITLE-ABS-KEY ("adopt*" OR "enact*" OR "adapt*" OR "apply" OR "applies" OR "application*" OR "innovat*" OR "uptake" OR "use" OR "usage" OR "using" OR "utiliz*")
28. TITLE-ABS-KEY ("income" OR "employ*" OR "compensat*" OR "earn*" OR "livelihood*" OR "pay" OR "payment*" OR "proceed" OR "proceeds" OR "profit*" OR "salar*" OR "harvest*" OR "productiv*" OR "income*" OR "revenue*" OR "wage*" OR "subsistence" OR "enterprise" OR "food secur*" OR "human nutrition" OR "on-farm" OR "off-farm" OR "labor" OR "labour" OR "value add*" OR "improv*" OR "soil fertility" OR "soil exhaustion" OR "socioeconomic*" OR “socio-economic*")
29. #27 OR #28
30. #15 AND #18 AND #26 AND #29

The impact of ruminant feed interventions on livelihoods among small scale and agro-pastoral livestock keepers in LMICs in Africa, Asia and Latin America: a scoping review

Search strategy for Dissertations and Theses Global (ProQuest)

###Searches performed May 24, 2019 (updated October 9, 2019)

####Number of records retrieved n=217

1. noft("American Samoa" OR "China" OR "Fiji" OR "Micronesia" OR "Indonesia" OR "Cambodia" OR "Kiribati" OR "Lao" OR "Marshall Islands" OR "Myanmar" OR "Mongolia" OR "Malaysia" OR "Nauru" OR "Philippines" OR "Papua New Guinea" OR "Korea" OR "Solomon Islands" OR "Thailand" OR "Timor-Leste" OR "Tonga" OR "Tuvalu" OR "Vietnam" OR "Vanuatu" OR "Samoa")
2. noft("Belize" OR "Bolivia" OR "Brazil" OR "Colombia" OR "Costa Rica" OR "Cuba" OR "Dominica" OR "Dominican Republic" OR "Ecuador" OR "Grenada" OR "Guatemala" OR "Guyana" OR "Honduras" OR "Haiti" OR "Jamaica" OR "St. Lucia" OR "Mexico" OR "Nicaragua" OR "Peru" OR "Paraguay" OR "El Salvador" OR "Suriname" OR "St. Vincent and the Grenadines" OR "Venezuela")
3. noft("Djibouti" OR "Algeria" OR "Egypt" OR "Iran" OR "Iraq" OR "Jordan" OR "Lebanon" OR "Libya" OR "Morocco" OR "West Bank" OR "Gaza" OR "Syrian Arab Republic" OR "Tunisia" OR "Yemen")
4. noft("Angola" OR "Benin" OR "Botswana" OR "Burkina Faso" OR "Burundi" OR "Cameroon" OR "Cape Verde" OR "Cabo Verde" OR "Central African Republic" OR "Chad" OR "Tchad" OR "Comoros" OR "Congo" OR "Cote d'Ivoire" OR "Ivory Coast" OR "Djibouti" OR "Equatorial Guinea" OR "Eritrea" OR "Ethiopia" OR "Gabon" OR "Gambia" OR "Ghana" OR "Guinea" OR "Kenya" OR "Lesotho" OR "Liberia" OR "Madagascar" OR "Malawi" OR "Mali" OR "Mauritania" OR "Mauritius" OR "Mozambique" OR "Namibia" OR "Niger" OR "Nigeria" OR "Réunion" OR "Rwanda" OR "Sao Tome and Principe" OR "Senegal" OR "Seychelles" OR "Sierra Leone" OR "Somalia" OR "South Africa" OR "Sudan" OR "Swaziland" OR "Swasiland" OR "Eswatini" OR "Tanzania" OR "Togo" OR "Uganda" OR "Western Sahara" OR "Zaire" OR "Zambia" OR "Zimbabwe" OR "Rhodesia")
5. noft("Afghanistan" OR "Bangladesh" OR "Bhutan" OR "India" OR "Maldives" OR "Nepal" OR "Pakistan" OR "Sri Lanka")
6. S1 OR S2 OR S3 OR S4 OR S5
7. noft("developing" OR "less-developed" OR "under-developed" OR "underdeveloped" OR "middle income" OR "low income" OR "underserved" OR "under served" OR "deprived" OR "poor")
8. noft("countr*" OR "nation*" OR "population*" OR "world")
9. S7 AND S8
10. noft("developing" OR "less-developed" OR "under developed" OR "underdeveloped" OR "low gdp" OR "low gnp" OR "low gross domestic" OR "low gross national")
11. noft("economy" or "economies")
12. S10 AND S11
13. noft("lmic" OR "lmics" OR "third world" OR "lami country" OR "lami countries" OR "transitional country" OR "transitional countries")
14. S9 OR S12 OR S13
15. S6 OR S14

16. noft("smallhold*" OR "small* hold* OR "small farm* OR "microfarm*" OR "micro-farm*" OR "pastoral" OR "family-run farm*" OR "family-owned farm*" OR "family-managed farm*" OR "agropastoral" OR "agro-pastoral" OR "ejido")
17. noft(("small-scale" OR "smallscale" OR "low-income" OR "subsistence" OR "semi-subsistence" OR "resource-poor" OR "resource-limited" OR " small-size*" OR "low-income" OR "peasant" OR "district*" OR "village" OR "local" OR household*) NEAR/3 ("farm*" OR "mixed-farm*" OR "agriculture" OR "producer*" OR "grower*" OR "agronomy" OR "husbandry"))
18. S16 OR S17
19. noft("multipurpose tree*" OR "multipurpose crop*" OR "fallow*" OR "total mixed ration*" OR "forag*" OR "fodder" OR "leguminous shrub*" OR "cut-and-carry" OR "tree lopping" OR "fodder")
20. noft("hay" OR “silage" OR "ensil*" OR “bale" OR “baling" OR "baler" OR “acetylation" OR “electrolysis" OR “esterification" OR "hydration" OR “hydrogenation" OR “iodination" OR “neutralization" OR “tanning" OR “hay-making")
21. noft("crop residue*" OR "roughage" OR "stover" OR “straw")
22. noft(("osmo*" OR “chemical" OR “acid*" OR “alkali*" OR “ammonia" OR “ion exchange" OR “sodium hydroxide") Near/2 (“treat*"))
23. noft(“chopping" OR “chemical treatment" OR “urea" OR “pulveriz*" OR “supplement*" OR “feed* block*")
24. S22 OR S23
25. S21 AND S24
26. S19 OR S20 OR S25
27. noft("adopt*" OR "enact*" OR "adapt*" OR "apply" OR "applies" OR "application*" OR "innovat*" OR "uptake" OR "use" OR "usage" OR "using" OR "utiliz*")
28. noft("income" OR "employ*" OR "compensat*" OR "earn*" OR "livelihood*" OR "pay" OR "payment*" OR "proceed" OR "proceeds" OR "profit*" OR "salar*" OR "harvest*" OR "productiv*" OR "income*" OR "revenue*" OR "wage*" OR "subsistence" OR "enterprise" OR "food secur*" OR "human nutrition" OR "on-farm" OR "off-farm" OR "labor" OR "labour" OR "value add*" OR "improv*" OR "soil fertility" OR "soil exhaustion" OR "socioeconomic*" OR “socio-economic*")
29. S27 OR S28
30. S15 AND S18 AND S26 AND S29
